# Immunogenicity and protective efficacy of an intranasal live-attenuated vaccine against SARS-CoV-2

**DOI:** 10.1016/j.isci.2021.102941

**Published:** 2021-08-04

**Authors:** Jun-Gyu Park, Fatai S. Oladunni, Mohammed A. Rohaim, Jayde Whittingham-Dowd, James Tollitt, Matthew D.J. Hodges, Nadin Fathallah, Muhsref Bakri Assas, Wafaa Alhazmi, Abdullah Almilaibary, Munir Iqbal, Pengxiang Chang, Renee Escalona, Vinay Shivanna, Jordi B. Torrelles, John J. Worthington, Lucy H. Jackson-Jones, Luis Martinez-Sobrido, Muhammad Munir

**Affiliations:** 1 Texas Biomedical Research Institute, Host-Pathogen Interactions and Population Health Programs, San Antonio, TX 78227, USA; 2 Division of Biomedical and Life Sciences, Faculty of Health and Medicine, Lancaster University, Lancaster LA1 4YG, UK; 3 Department of Virology, Faculty of Veterinary Medicine, Cairo University, Giza, 12211, Egypt; 4 Faculty of Applied Medical Sciences, Department of Medical Laboratory Technology, Immunology Group, King Abdul Aziz University, Jeddah 80200, Saudi Arabia; 5 Faculty of Applied Medical Sciences, Department of Medical Laboratory Technology, Microbiology Group, King Abdul Aziz University, Jeddah 80200, Saudi Arabia; 6 Faculty of Medicine, Al Baha University, Al Baha 77388, Saudi Arabia; 7 The Pirbright Institute, Woking GU24 0NF, UK

**Keywords:** immunology, virology

## Abstract

Global deployment of an effective and safe vaccine is necessary to curtail the coronavirus disease 2019 (COVID-19) caused by severe acute respiratory syndrome coronavirus 2 (SARS-CoV-2). Here, we evaluated a Newcastle disease virus (NDV)-based vectored-vaccine in mice and hamsters for its immunogenicity, safety, and protective efficacy against SARS-CoV-2. Intranasal administration of recombinant (r)NDV-S vaccine expressing spike (S) protein of SARS-CoV-2 to mice induced high levels of SARS-CoV-2-specific neutralizing immunoglobulin A (IgA) and IgG2a antibodies and T-cell-mediated immunity. Hamsters immunized with two doses of vaccine showed complete protection from lung infection, inflammation, and pathological lesions following SARS-CoV-2 challenge. Importantly, administration of two doses of intranasal rNDV-S vaccine significantly reduced the SARS-CoV-2 shedding in nasal turbinate and lungs in hamsters. Collectively, intranasal vaccination has the potential to control infection at the site of inoculation, which should prevent both clinical disease and virus transmission to halt the spread of the COVID-19 pandemic.

## Highlights

• Vaccine induces high levels of neutralizing Abs and T-cell-mediated immunity
• Vaccine ameliorates lung inflammation and pathology in hamster induced by SARS-CoV-2
• The SARS-CoV-2 remains undetectable in lungs and nasal turbinates of vaccinated hamster
• Two doses of intranasal vaccine show complete protection against SARS-CoV-2 challenge

## Graphical abstract

**Figure:**
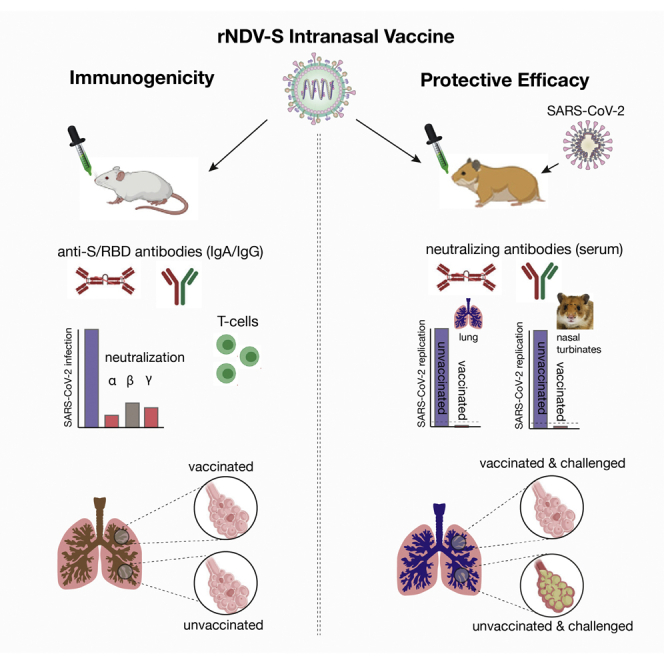

## Introduction

Coronavirus disease 2019 (COVID-19), caused by the severe acute respiratory syndrome coronavirus 2 (SARS-CoV-2), emerged in a seafood market in Wuhan, China during late 2019 and was linked to pneumonia-associated illness, which can culminate in respiratory failure (Guan et al., 2020). COVID-19 can also manifest in the form of cardiovascular and gastrointestinal pathologies and induce a hyperinflammatory syndrome (Cheung et al., 2020; Mao et al., 2020; Wichmann et al., 2020). People of older age (>65 years) and with existing comorbidities such as obesity, diabetes, immunosuppression, and hypertension, among others, are highly vulnerable and may bear severe COVID-19 clinical symptoms (Zhou et al., 2020). COVID-19 has currently overwhelmed health care systems around the world with >75 million infections resulting in hundreds of thousands of deaths, with a case-fatality rate of approximately 4% (WHO, Coronavirus Disease [COVID-19] Dashboard. https://covid19.who.int/). The enormous impact of COVID-19 directly on lives and its socioeconomic effect on the entire human population require the development of a vaccine that not only reduces the severity of the SARS-CoV-2 infection but also curbs virus transmission.

SARS-CoV-2 is a positive-sense, single-stranded RNA virus with a genome of approximately 30,000 nucleotides. Among all viral proteins, the spike (S) proteins play critical roles in viral entry. In addition, S protein contains several crucial B cell and T cell epitopes (Letko et al., 2020; Walls et al., 2020) and remains the main target for neutralizing antibodies (NAbs). Therefore, it serves as a promising vaccine target against coronaviruses, including SARS-CoV-2.

Since the release of the SARS-CoV-2 genome sequence, a range of vaccine platforms were repurposed to principally target the S protein of SARS-CoV-2 (Graham, 2020). Currently, a number of candidate SARS-CoV-2 vaccines are either under development, including viral vector, inactivated virion, and recombinant protein-based vaccines, or being authorized for emergency-only use such as lipid nanoparticle encapsulated mRNA vaccines (Zhu et al., 2020; Logunov et al., 2020). Although each of these vaccine platforms present their merits and limitations, vector-based vaccines possess enormous potential at eliciting strong and long-lasting adaptive immunity inducing antibody (Ab) responses (Zhu et al., 2020; Logunov et al., 2020). Most of the currently developed SARS-CoV-2 vector-based vaccines adopt the intramuscular route of administration; however, it has been demonstrated that the intranasal route offers superior induction of local mucosal immunity and protection against SARS-CoV-2 (Hassan et al., 2020).

Indeed, owing to the nature of the virus biology, the Newcastle disease virus (NDV) vector offers multifaceted and superior advantages for intranasal immunization. As an avian virus, NDV is fully attenuated in human and nonhuman primates and causes flulike illness with short-lived conjunctivitis in humans (Bukreyev and Collins, 2008). Several strains of NDV have been proved to be safe and are used extensively as oncolytic agents in humans (Bukreyev and Collins, 2008). NDV is genetically stable and can tolerate insertion of foreign transgenes of approximately 5 Kb into its genome without compromising viral replication (Bukreyev and Collins, 2008). Notably, humans typically lack preexisting immunity against NDV compared with currently applied human viral vectors such as the human adenovirus (Ad), measles virus (MeV), modified vaccinia Ankara (MVA), or vesicular stomatitis virus (VSV). Owing to its tendency to replicate in the respiratory tract, NDV has also been shown to induce local mucosal immunity, offering an additional advantage via potential inhibition of viral shedding. Importantly, NDV-based vaccines can be produced in chicken embryonated eggs or in US Food and Drug Administration (FDA)-approved Vero cell lines using protocols and infrastructure currently being applied to current influenza virus vaccines. Given these and other features, apathogenic strains of NDV have been proposed as live attenuated vaccines (LAVs) against multiple viruses including influenza (Ge et al., 2007; DiNapoli et al., 2010a), SARS-CoV (Bukreyev et al., 2005), human immunodeficiency virus (Carnero et al., 2009), human parainfluenza (Hurwitz et al., 1997; Haller et al., 2000), rabies (Ge et al., 2011), Nipah (Kong et al., 2012), Rift Valley fever (Kortekaas et al., 2010), and Ebola (Yang et al., 2008; DiNapoli et al., 2010b; Yoshida et al., 2019). Notably, NDV-based vaccines have been shown to be safe and effective in multiple animal models including mice (Ge et al., 2007), dogs (Ge et al., 2011), pigs (Kong et al., 2012), cattle (Khattar et al., 2010), African green and rhesus monkeys (Bukreyev et al., 2005; DiNapoli et al., 2010a), and ultimately humans (Ockert et al., 1996; Pecora et al., 2002; Karcher et al., 2004).

We have recently developed an NDV-based SARS-CoV-2 vaccine encoding a human codon-optimized full-length wild-type spike (S) protein of SARS-CoV-2 (rNDV-S) using a reverse genetic approach (Rohaim and Munir, 2020). The rNDV-S successfully replicated in chicken embryonated eggs and cultured cells to levels comparable to the recombinant wild-type NDV (rNDV-WT) (Rohaim and Munir, 2020). Here we tested the immunogenicity and safety of this rNDV-S-based LAV in mice and advanced our studies to assess protective efficacy in hamsters. Two intranasal doses of the rNDV-S vaccine induced robust systemic humoral and cell-mediated immune responses in mice and fully protected hamsters against lung infection, inflammation, and pathology following SARS-CoV-2 challenge. Intranasal administration of two doses of rNDV-S induced high levels of SARS-CoV-2 NAbs and anti-SARS-CoV-2 immunoglobulin A (IgA) and IgG2a and conferred complete protection against SARS-CoV-2 infection. Importantly, double dose of rNDV-S significantly reduced viral shedding into nasal turbinate and lungs of hamsters. Altogether, intranasal immunization of rNDV-S has the potential to control SARS-CoV-2 infection at the site of inoculation, which should prevent both virus-induced disease and transmission, representing an excellent option for the prevention of SARS-CoV-2 infection and associated COVID-19 disease.

## Results

### Intranasal vaccination with rNDV-S successfully establishes a viral load with no accompanying adverse pathology

In order to explore the potential for an intranasal live-attenuated vector vaccine against SARS-CoV-2, we engineered rNDV-S (i.e., avian orthoavulavirus 1, AOaV-1) encoding a human codon-optimized SARS-CoV-2 full-length and wild-type S glycoprotein gene, including the ectodomain, transmembrane domain, and cytoplasmic domain. The S gene was cloned into a preoptimized gene junction between the phosphoprotein and matrix gene of NDV. As we demonstrated previously (Rohaim and Munir, 2020), rNDV-S replicated comparably to rNDV-WT in both cell culture and avian eggs and migrates within cells independently of exogenous trypsin, proposing a competitive vaccine candidate.

**Figure 1:**
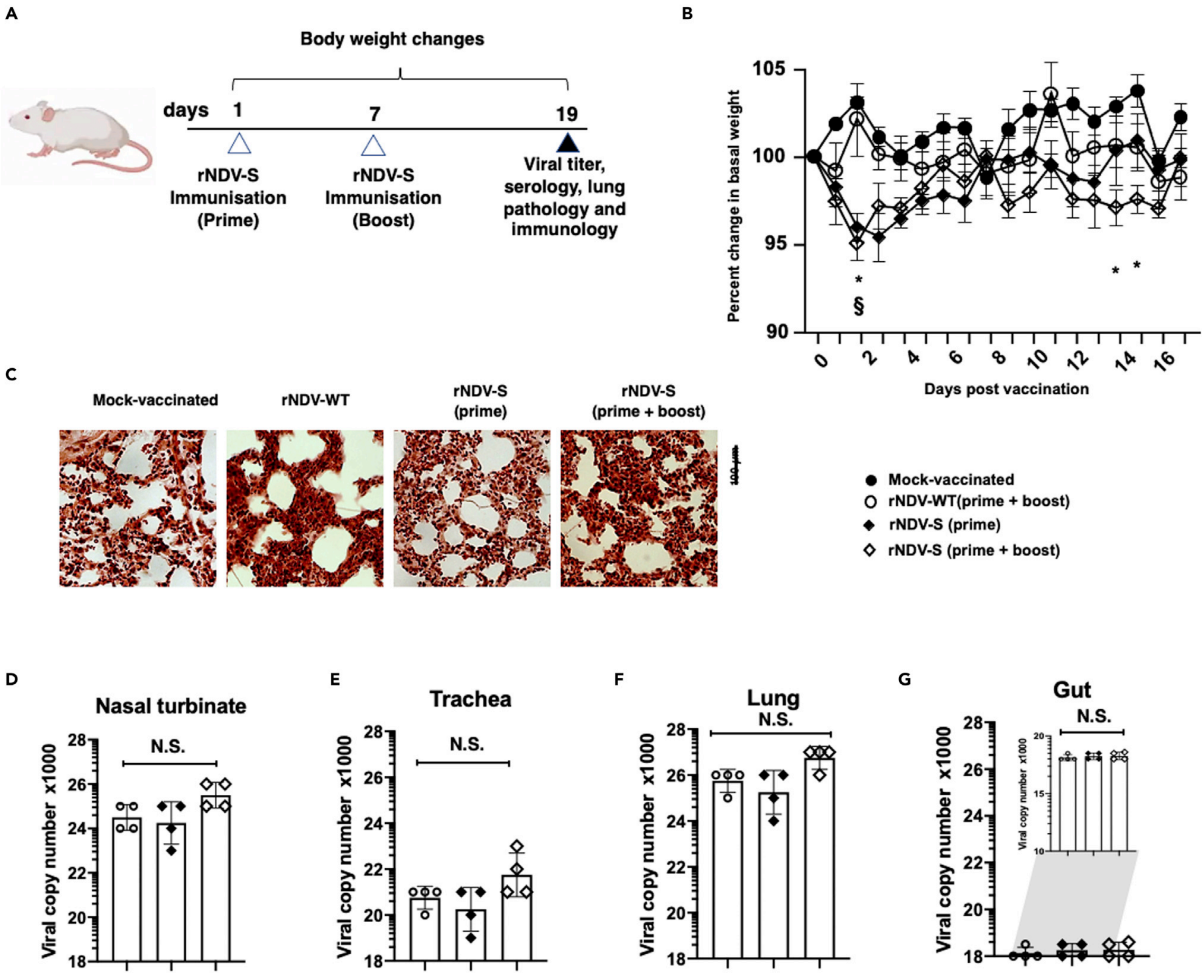
No adverse pathology results following rNDV-S intranasal vaccination (A) Experimental model for the prime and prime + boost intranasal vaccination of mice. (B) Percentage weight change of mice following instillation of PBS or indicated rNDV constructs over the experimental time course. (C) Representative images of right lung following H&E staining. (D–G) Viral replication of intranasally administered rNDV in nasal turbinate (D), trachea (E), lung (F), and gut (G). Data (n = 4–5 mice/group); ∗, P < 0.05; and NS, nonsignificant between naive and vaccinated groups; error bars represent SE of means via repeated t test or ANOVA with Dunnett's posttest.

To assess the safety and immunogenicity of our rNDV-S vaccine, groups of 12-week-old BALB/c mice were immunized by intranasal inoculation with 10^6^ PFU of test vaccine rNDV-S or wild-type NDV (rNDV-WT) or were mock-vaccinated with phosphate buffer saline (PBS). The rNDV-WT and one rNDV-S group of mice received a booster dose of 10^6^ PFU of rNDV-WT or rNDV-S, respectively, a week later, whereas other groups were mock-boosted (Figure 1A). All animals were euthanized on day 19 postvaccination for safety assessment, as well as to assess pathological and immunological responses. Mice were monitored daily for weight loss, health status, and feed intake. Initial starting weights did not significantly differ in any experimental group prior to treatment (Figure 1B), and there was no significant alteration in the percentage of basal weight of mice in groups immunized with rNDV-S and the control mock-vaccinated group across the time course of the experiment (Figure 1B). The first dose of rNDV-S induced a small but significant reduction in basal weight at 2 and 3 days postvaccination (DPV), but weights returned to comparable levels to mock-immunized mice on the following day and for the rest of the time course (Figure 1B). A reduction in weight was also seen at 14 and 15 DPV in the rNDV-S (prime + boost) group, but weights also quickly returned to mock levels for the rest of the study. Analysis of daily chow intake revealed no significant differences in daily chow consumption among groups (Figure S1A). Mice were scored daily assessing movement, coat, temperature, eyes, and signs of hunching on a 14-point scale. No adverse clinical disease signs were observed with an indicated daily score of “0” in the duration of experiments. Following the end of the experiment at day 19 postvaccination, the gross and histopathological assessment of the right lung lobe showed no significant lesions in any of the treated groups (Figures S1B and 1C). In addition, to confirm that intranasally delivered rNDV-S replicated in immunized mice, we examined the expression of the matrix (M) gene of NDV in various tissues at 19 DPV using qRT-PCR. Results indicated a high number of NDV copies, and a comparable replication of rNDV-S and rNDV-WT was observed in the respiratory tract (nasal turbinate, trachea, and lungs) (Figures 1D–1F). However, comparatively reduced replication was observed in the gut of mice vaccinated with rNDV-S or rNDV-WT compared with the respiratory tract (Figure 1G). Collectively, these data indicate that rNDV-S intranasal vaccination produces no adverse pathology in the tissues examined and the rNDV-S vaccine replicated significantly in the target tissues comparable to the rNDV-WT.

### Intranasal immunization with rNDV-S elicits potent S-protein binding Abs and NAbs in mice

**Figure 2:**
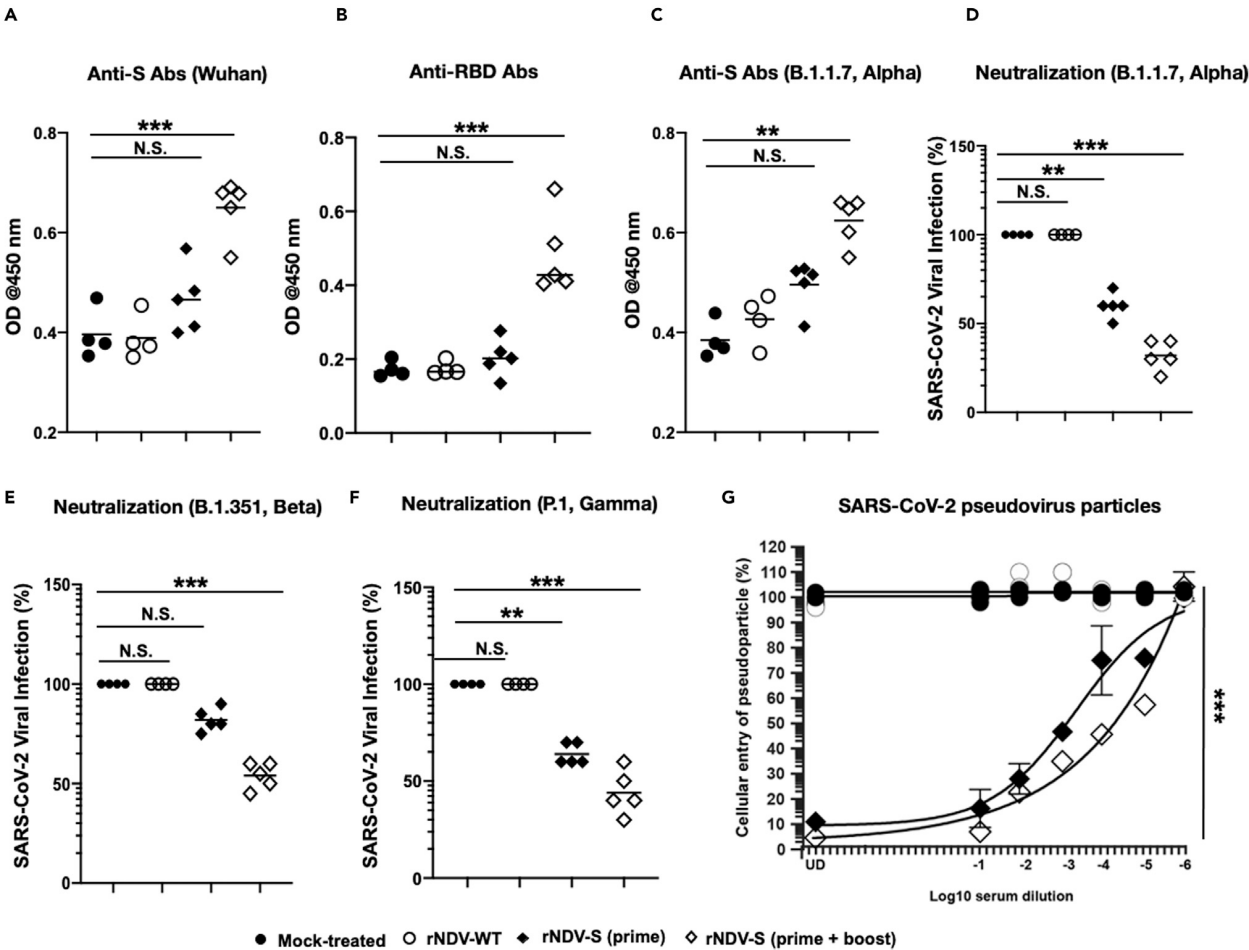
Intranasal administration of rNDV-S elicits titers of binding and NAbs in mice (A) S-specific serum IgG titers measured by ELISA. Sera from animals at 19 days after prime and after prime + boost were isolated and used in the ELISA against a recombinant trimeric S protein. Sera from mock-vaccinated mice and mice vaccinated with rNDV-WT were used as control. (B) Corresponding sera from all group of mice were used to detect Abs against RBD of spike protein. (C) Sera from all group of mice were used to detect Abs against full-length spike protein from B117 mutant strain of SARS-CoV-2. (D–F) (D) Sera samples from all groups were used to measure neutralization of SARS-CoV-2 B.1.1.7 (D), B1.1351 (E), and P.1 (F) variants. Sera from prime group of mice that were vaccinated with rNDV-S prime only or after prime + boost group of mice showed marked virus neutralization compared with sera from mock-treated or mice vaccinated with rNDV-WT. (G) Lentiviruses expressing the S protein of SARS-CoV-2 were used to perform a pseudoparticle entry inhibition assay. Sera samples collected from mice after prime or after prime + boost inhibited the entry of lentiviral pseudoparticles compared with sera collected from mice vaccinated with rNDV-WT or mock-vaccinated. Data (n = 4–5 mice/group); ∗, P < 0.05; ∗∗, P < 0.01; or ∗∗∗, P < 0.005 between naive and vaccinated groups; error bars represent SE of means via repeated T test or ANOVA with Dunnett's.

To evaluate the immunogenicity of rNDV-S, sera samples collected at 19 days postvaccination were used to evaluate total IgG antibody titers using ELISAs and levels of neutralizing antibodies using microneutralization and pseudoparticle entry inhibition assays. To detect antispike serum Abs, full-length spike (S) protein or recombinant Spike protein receptor binding domain (RBD), expressed using the Drosophila S2 cell system, were used to coat ELISA plates. After prime immunization only, the sera from mice immunized with rNDV-S contained S-protein-specific Abs, which were significantly increased with the boosting dose (Figure 2A). In comparison, no S-protein-specific Abs were detected in sera from the mice immunized with rNDV-WT or the mock-immunization (Figure 2A). Next, we assessed the levels of total IgG responses against purified RBD. The rNDV-S only induced significantly high levels of anti-RBD-specific IgG in the prime + boost immunized group when compared with mock-immunized or rNDV-WT-immunized mice (Figure 2B). Using recombinant S protein from the B117 mutant, the data showed a significant binding of antibodies in prime + boost group compared with prime only, rNDV-WT, and the mock-vaccinated mice (Figure 2C). We next functionally characterized serum Ab responses by lentiviral entry inhibition and focal-reduction neutralization tests (Schmidt et al., 2020; Park et al., 2021). As expected, compared with mice mock-immunized or immunized with rNDV-WT, a significant level of neutralization was observed in sera collected from rNDV-S-immunized mice against SARS-CoV-2 B.1.1.7 (Alpha) (Figure 2D), B.1.351 (Beta) (Figure 2E), and P.1 (Gamma) (Figure 2F) variants. Although neutralization was observed in sera collected from both primed and prime + boosted mice, the latter was more significant in generating NAbs. Correspondingly, serum from mock- or rNDV-WT-vaccinated mice did not inhibit the entry of pseudoviral particles (Figure 2G). In contrast, serum from rNDV-S prime or rNDV-S prime + boost groups significantly inhibited the entry of the pseudoviral particles. Overall, rNDV expressing SARS-CoV-2 S protein elicited high titters of protein binding and virus NAbs in mice, especially when a prime + boost regime was utilized.

### Intranasal vaccination with rNDV-S does not induce overt myeloid inflammatory responses in the lungs, pleural cavity, or systemically

We next analyzed systemic and local immunological responses to determine whether innate and adaptive responses were beyond the basal response induced by rNDV-WT. We did not observe significant alterations in cellularity of the spleen, used as a surrogate marker of systemic inflammation (Figure S2A). We also did not observe significant changes in the rNDV-S prime + boost group percentages of myeloid subsets examined, including neutrophils, monocytes, inflammatory monocytes, or eosinophils (Figure S2B). However, we observed a significant increase in the percentage of dendritic cells (DCs) in the spleen of rNDV-WT immunized animals as compared with the mock vehicle control, and although no significant increase was seen in the rNDV-S prime immunized group, a similar but nonsignificant trend in DC levels was seen in murine spleen after prime + boost rNDV-S immunization (Figure S2B). Despite no significant morphological changes in the cellular architecture in the lung (Figure 1C), we did observe a significant increase in the cellularity of the lung in the rNDV-S (prime + boost)-immunized group, as compared with all other experimental groups studied (Figure S2C). However, when we further examined the myeloid immune subsets, we did not observe significant differences in neutrophils, Ly6C− monocyte/macrophages, alveolar macrophages, and eosinophils as compared with the mock vehicle control group. A significant decrease in the Ly6c+ monocyte/macrophage subset was observed in the rNDV-S (prime + boost) group (Figure S2D). Interestingly and similar to the results in the spleen, we only observed a significant increase in the total DC population in the lung of mice receiving the rNDV-WT vaccine as compared with mock-vaccinated group (Figure S2D).

To investigate further whether a local inflammatory response occurred following intranasal vaccination, we assessed the immune response within the pleural cavity. Our results indicate that there were no statistically significant increases in total CD45^+^ hematopoietic cells, neutrophils, eosinophils, monocytes, and F4/80^lo^MHCII^hi^ myeloid cells within either pleural lavage fluid (Figures S3A–S3F) or pericardial adipose tissue (Figures S3G–S3K). However, although not significant, a trend for higher amounts of F4/80^hi^MHCII^lo^ pleural cells was observed in rNDV-S prime + boost group (Figure S3F). Collectively, these data suggest that there is no lasting abhorrent myeloid inflammatory response generated at the site of vaccination or systemically following intranasal delivery of rNDV-S as compared with the mock-vaccinated mice, but a residual myeloid antigen-presenting cell response is apparent, which may have successfully driven adaptive immunity and thus, potential protection.

### Intranasal vaccination with rNDV-S drives T cell IFNγ responses both in the lung and systemically

**Figure 3:**
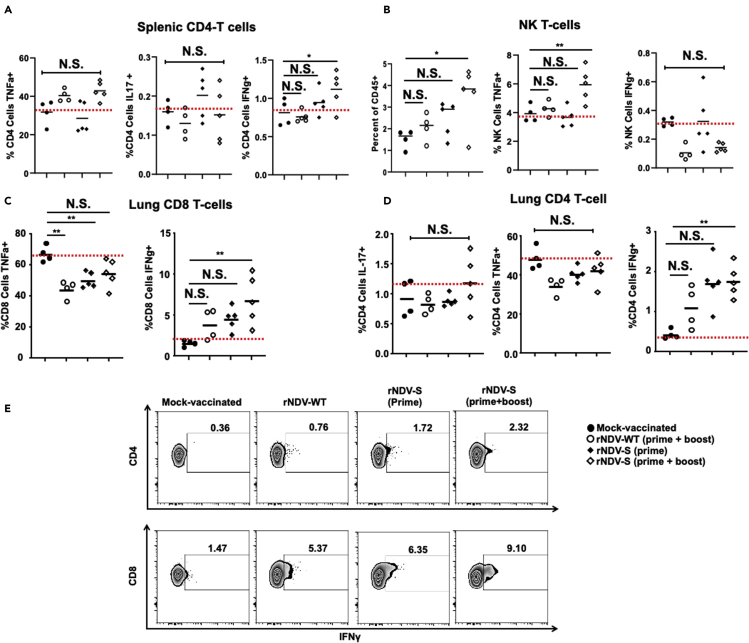
Vaccination with rNDV-S drives both CD4 and CD8 T cell cytokine responses Flow cytometric analysis of cytokine responses to SARS-CoV-2 S protein from splenic CD4 (A) and NK (B) T cell populations and lung CD4 (C) and CD8 (D) T cell populations from mice on day 19 following instillation of mock (PBS) or indicated rNDV constructs intranasally on days 0 and 7. Red dashed line indicates baseline cytokine response to PMA/ionomycin without antigen (E). Representative T cell flow cytometry plots of lung cytokine production of (C) and (D). Data (n = 4–5 mice/group); ∗, P < 0.05; ∗∗, P < 0.01; or ∗∗∗, P < 0.005 between naive and vaccinated groups; error bars represent SE of means via ANOVA with Dunnett's posttest.

We next examined T cell subsets, asking what cytokine responses were produced against the purified full-length S protein both systemically and locally at the vaccination site. Systemically, within the spleen, there was no alteration in the percentage of splenic CD4+ or CD8+ T cell subsets in any of the experimental groups studied (Figure S4A). Beyond a trend for increased means in all vaccinated groups, we also did not see a significant increase in splenic CD8+ T cell TNF^+^ or CD8+ T cell interferon gamma positive (IFNγ^+^) cell populations in response to restimulation in any of the groups studied (Figure S4B). Conversely, splenic CD4+ T cell IFNγ^+^ (Figure 3A) and NK T cell TNF^+^ (Figure 3B) were significantly increased in the rNDV-S boosted group, indicating a systemic response to SARS-CoV-2 S antigen following vaccination with rNDV-S.

Locally, within the lung homogenate, a significant decrease in CD4+ T cells and an increase in CD8+ T cell percentage was observed following vaccination with all NDV-containing groups (Figure S4C). Looking at cytokine responses we saw a significant reduction, below the baseline antigen-free stimulation, in CD8+ T cell tumor necrosis factor alpha (TNF-α) production in both the NVD-WT and NVD-S groups, and a significant increase in IFNγ production in the rNDV-S-boosted group as compared with the mock vehicle group (Figures 3C and 3E). However, despite an overall decrease of CD4 T cell percentage and no alteration in CD4+ T cell subpopulations producing interleukin-17 (IL-17) or TNF-α, a significant increase in CD4+ T cell IFNγ^+^ (6.4-fold) was observed also in the rNDV-S-boosted group as compared with the mock-vaccinated group (Figures 3D and 3E). Collectively, these data indicate no hyperinflammation but both a systemic and local IFNγ T cell cytokine response to viral antigen following intranasal vaccination with rNDV-S.

### Intranasal vaccination with rNDV-S drives NAb responses specifically targeting SARS-COV-2 S protein

**Figure 4:**
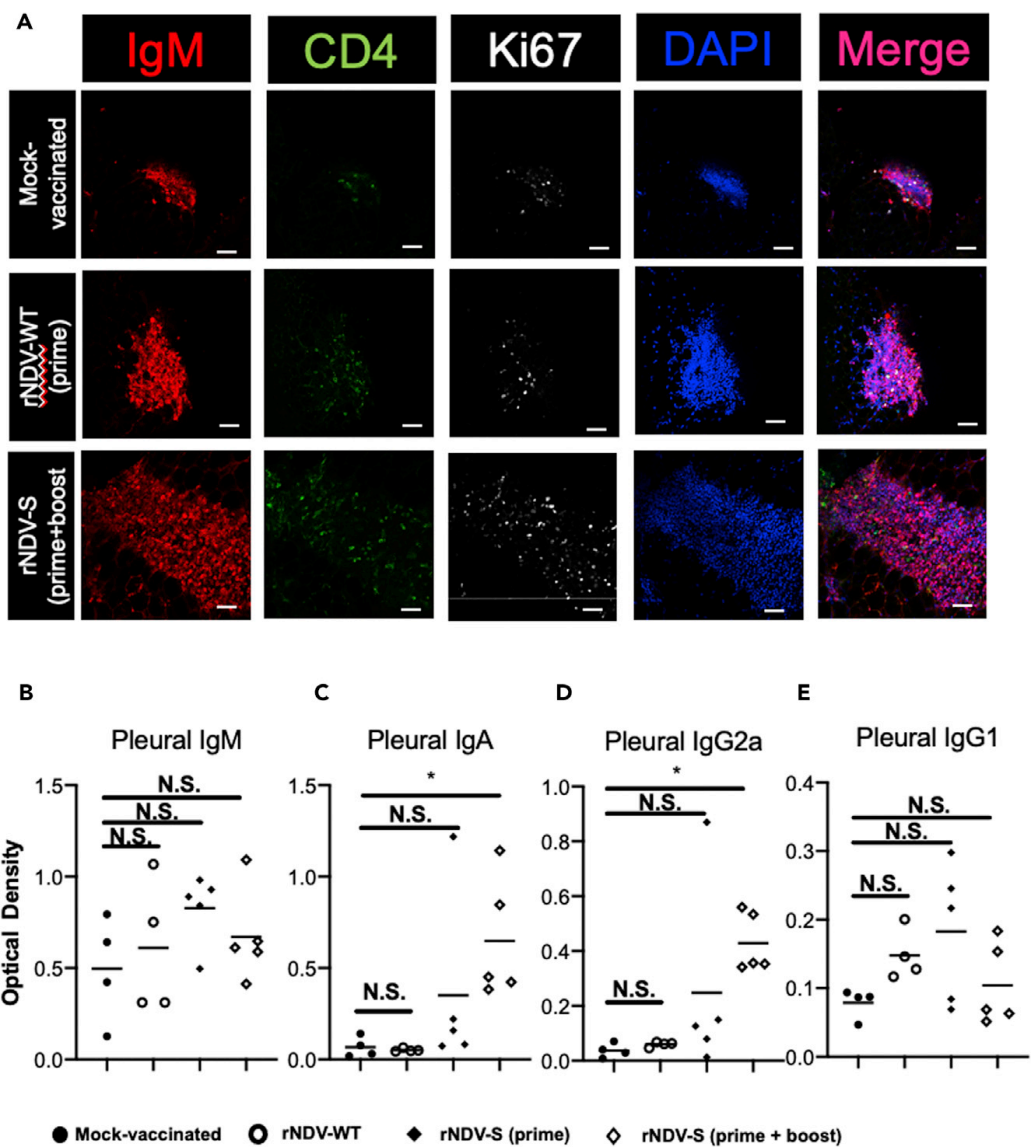
Fat-associated lymphoid clusters (FALCs) expand within the pericardium and class switched Abs are present within the pleural fluid following vaccination Expansion of fat-associated lymphoid clusters within the thoracic cavity in response to rNDV-S. (A) Whole-mount immunofluorescence imaging of the murine mediastinum on day 19 following instillation of PBS or indicated rNDV constructs, intranasally, on days 0 and 7. IgM (B), IgA (C), IgG2a (D), and IgG1 (E) Abs against SARS-CoV-2 spike protein receptor binding domain present within pleural fluid on day 19 following instillation PBS or indicated NDV construct i.n. on day 0 and day 7. Data (n = 4–5 mice/group); ∗, P < 0.05; ∗∗, P < 0.01 between mock and vaccinated groups; N.S. = nonsignificant; error bars represent SE of means via one-way ANOVA with Sidak's posttest.

Fat-associated lymphoid clusters (FALCs) are sites of local Ab production within the pericardium and mediastinum, known for their early response to intranasal challenge (Jackson-Jones et al., 2016). Using whole-mount immunofluorescence staining, we were able to detect an expansion of FALCs within the mediastinum at 19 DPV in the rNDV-S-boosted group (Figures 4A and S5A). Within FALCs of mice receiving rNDV-S boost, and supporting an earlier response, there was a significant increase in the level of Ki67^+^ proliferation, representative of cellular division (Figures S5B and S5C). Although not reaching significance, a trend for increased percentage area of CD4 was seen, suggesting a local accumulation of CD4 T cells within the mediastinum (Figure S5D). No increase in the percentage area of immunoglobulin M (IgM) (B cell marker) was seen within the pericardium at day 19 DPV (Figure S5E).

IgM is the first polyclonal Ab induced during an immune response, and following induction of adaptive immunity B cells, IgM class switches to other Ab subclasses defined by the cytokine milieu. As FALCs are the site from which Abs detected in pleural fluid are produced, we next confirmed the local activation of B cells by assessing the presence of Abs against SARS-CoV2 S RBD within the pleural fluid. Within the pleural fluid no antigen-specific IgM was detected (Figure 4B); however, a significant increase in both RBD-specific IgA (Figure 4C) and IgG2a (Figure 4D), but not IgG1 (Figure 4E), levels was present within the pleural fluid of mice in the rNDV-S-boosted group compared with control mice and those receiving rNDV-WT. These data suggest that B cells producing the Abs had class switched and is consistent with the T-cell-derived IFNγ^+^ detected upon restimulation, driving a switch to IgA and/or IgG2a (Figures 4C and 4D). We next determined the number of B cells present within the pleural lavage fluid and digested pericardium and did not observe significant differences in the numbers of B1a, B1b, or B2 cells present within the pleural lavage fluid or pericardium when comparing rNDV-S-boosted with mock control mice (Figure S6). Furthermore, we also did not detect a significant increase in pericardial B cell proliferation (Figures S7A and S7B), suggesting that Abs detected within the pleural fluid may have been secreted earlier or resulted from an ongoing systemic B cell response within primary lymphoid organs. To further support this, systemic anti-full-length spike and anti-RBD Ab responses were observed, which neutralized SARS-CoV-2 (Figure 2).

### Intranasal vaccination with rNDV-S produces no adverse pathology in hamsters

**Figure 5:**
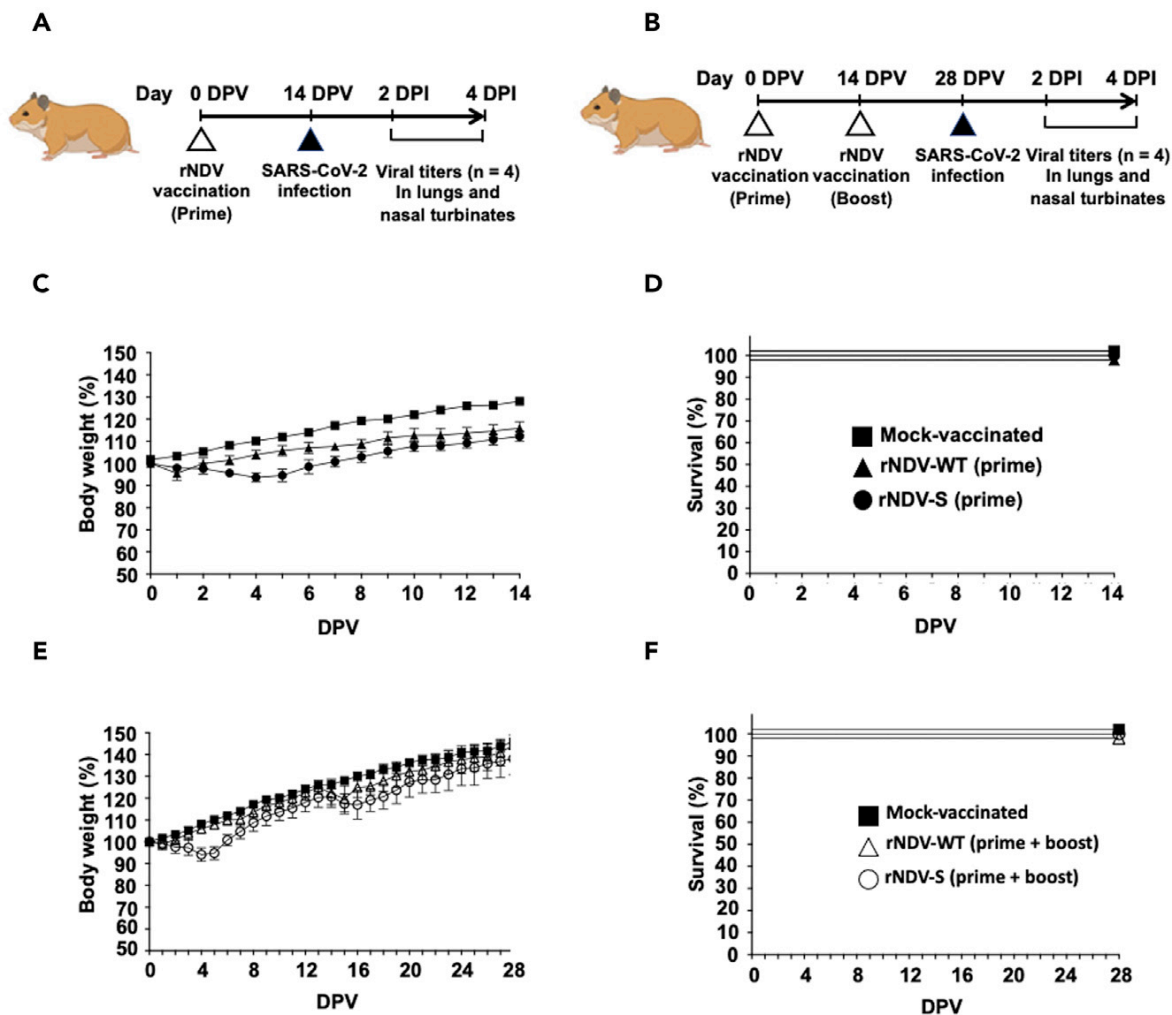
Safety of rNDV-S in golden Syrian hamsters (A and B) **Schematic representation of the vaccination approach:** golden Syrian hamsters (n = 8/group) were mock (PBS)-vaccinated or vaccinated intranasally with 1 × 10^6^ PFU of rNDV-WT or rNDV-S using a prime (A) or a prime + boost (B) vaccination regimen. After vaccination, hamsters were challenged at 14 (prime, (A) or 28 (prime + boost, **b**) DPV with 2 × 10^4^ PFU of SARS-CoV-2 and sacrificed at 2 and 4 DPV for gross lung pathology, viral titers, H&E staining, and histopathology. (C and D) **Morbidity and mortality of rNDV-S in prime vaccinated hamsters:** Body weight changes (C) and survival (D) were evaluated at the indicated DPV with a single dose of rNDV-WT or rNDV-S. Mock (PBS)-vaccinated hamsters were used as control. (E and F) **Morbidity and mortality of rNDV-S in prime + boost vaccinated hamsters:** body weight changes (E) and survival (F) were evaluated at the indicated DPV with two doses of rNDV-WT or rNDV-S. Mock (PBS)-vaccinated hamsters were included as control. Error bars represent standard deviations (SD) of the mean for each group of hamsters.

After establishing the basis of immunological and safety profiles in mice, we next attempted to evaluate the safety of intranasal rNDV-S vaccination in golden Syrian hamsters, which have the advantage of being susceptible to SARS-CoV-2 infection. A total of n = 8 hamsters in each group were mock (PBS) vaccinated or vaccinated with 1 × 10^6^ PFU of rNDV-WT or rNDV-S once (prime, Figure 5A) or twice in a 2-week interval (boosted, Figure 5B). Body weight was measured daily for 14 (prime) or 28 (boosted) DPV. Prime vaccination with rNDV-S resulted in no apparent clinical disease except slight (<5%) body weight loss at 5 DPV (Figure 5C) and no mortality (Figure 5D). Boosted vaccination also induced less than 10% of body weight loss by 5 DPV (Figure 5E), with all the animals surviving rNDV-S vaccination (Figure 5F). Vaccination with rNDV-WT resulted in body weight loss only one day after vaccination in either the prime or boosted vaccinated hamsters (Figures 5C and 5E). These results demonstrate that vaccination of golden Syrian hamsters with 1 × 10^6^ PFU of rNDV-S is safe without lasting significant changes in body weight, with all the animals surviving intranasal rNDV-S vaccination.

### Intranasally administered rNDV-S induces Ab and NAb responses in hamsters

**Figure 6:**
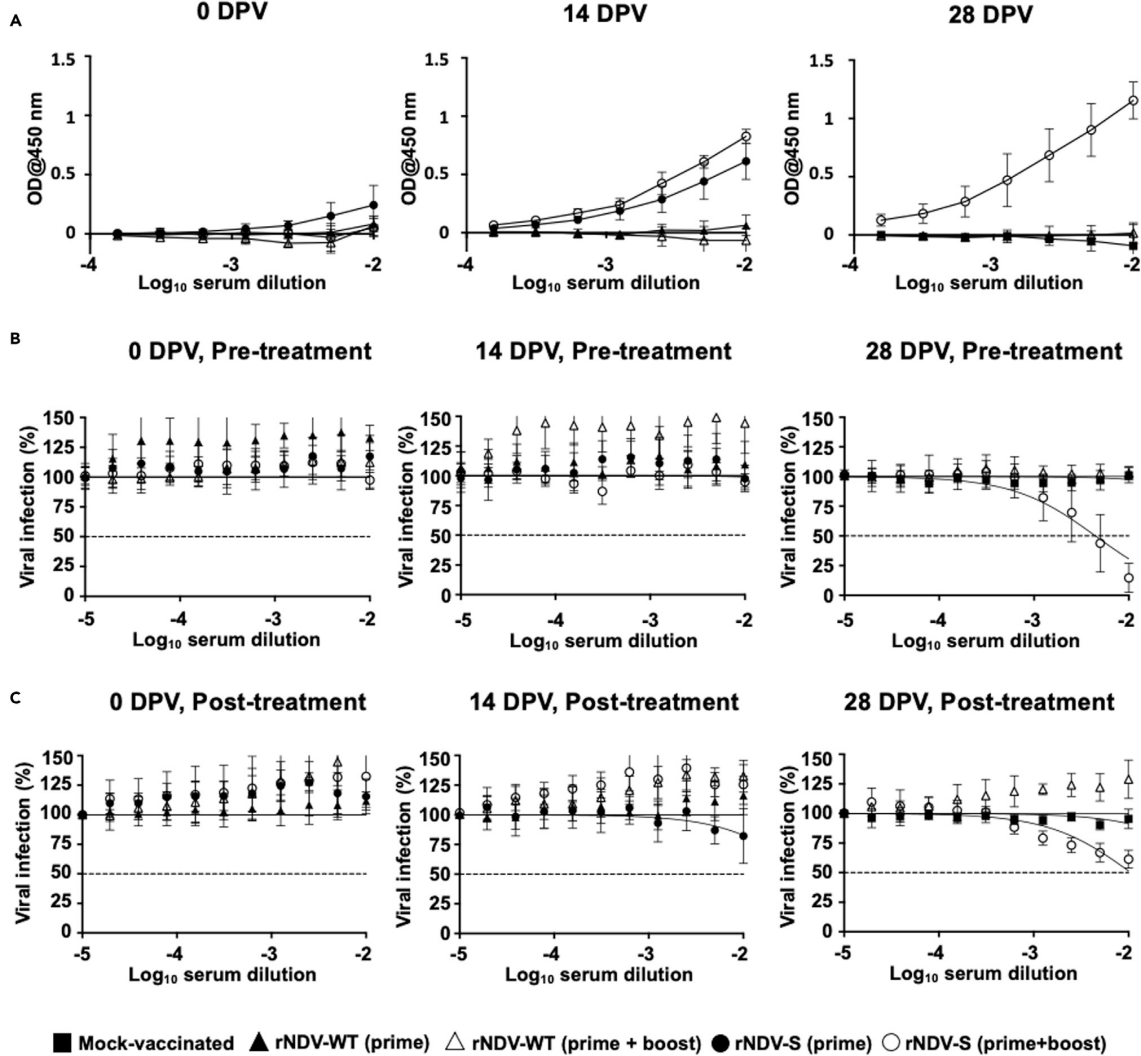
Total and NAbs in serum of golden Syrian hamsters vaccinated with rNDV-S (A) **ELISA:** levels of total S binding Abs in sera from hamsters vaccinated with rNDV-S at 0, 14, and 28 DPV. (B) **Neutralization assays, pretreatment conditions:***in vitro* neutralizing activity of hamster serum samples against SARS-CoV-2 at 0, 14, and 28 DPV. (C) **Neutralization assays, posttreatment conditions:***in vitro* neutralizing activity of hamster sera against SARS-CoV-2 at 0, 14, and 28 DPV. Virus neutralization assays were quantified using ELISPOT and the percentage of infectivity calculated using sigmoidal dose-response curves (B and C). Mock-infected cells and SARS-CoV-2 infections in the absence of serum were used as internal controls (B and C). Dotted line indicates 50% neutralization (B and C). Data were expressed as mean and SD.

Serum samples collected at 0, 14, and 28 DPV from experimental hamsters were analyzed for Ab and NAb responses against SARS-CoV-2. Total levels of Abs against SARS-CoV-2 were evaluated by ELISA, whereas NAbs were evaluated by PRNT assay using SARS-CoV-2. Sera from prime vaccinated hamsters (14 DPV) were able to react with extracts from SARS-CoV-2-infected cell homogenates, but not from mock-infected cell homogenates (Figure 6A). Notably, levels of total Abs against SARS-CoV-2 were higher in sera from boosted rNDV-S-vaccinated hamsters (28 DPV) (Figures 6A, S8, and S9). In terms of NAb responses, only sera (starting from 1:100 dilution) from hamsters receiving boosted vaccination (28 DPV) presented NAbs in the PRNT assay in both pretreatment (Figure 6B) and posttreatment (Figure 6C) conditions. Moreover, until 28 DPV, the NAb remained under the limit of detection (Table S1). These results indicate that boosting is required to induce robust NAb responses against SARS-CoV-2 upon vaccination with rNDV-S in hamsters.

### Protection efficacy of rNDV-S against SARS-CoV-2 infection in hamsters

**Figure 7:**
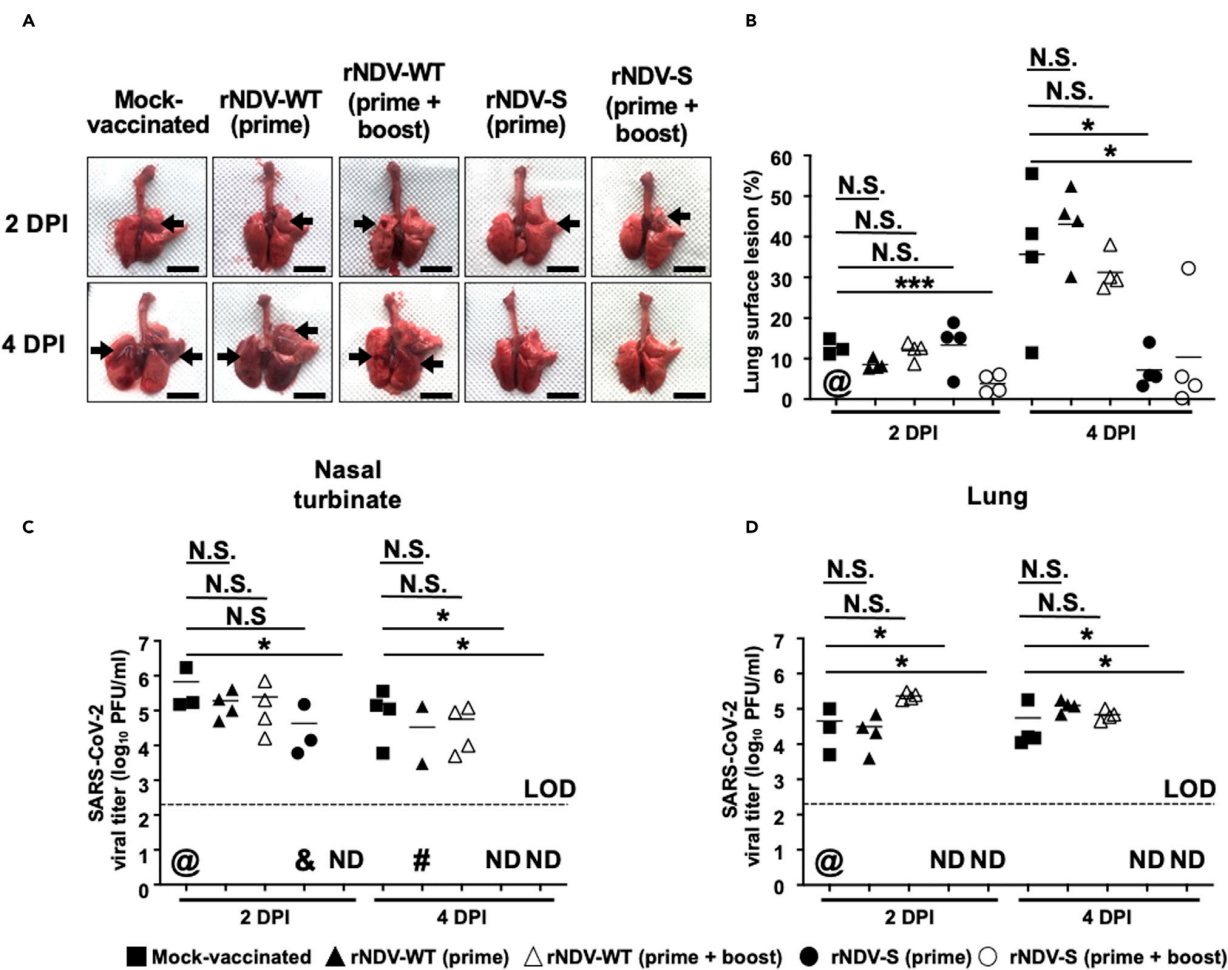
Gross lung pathology and SARS-CoV-2 titers in challenged golden Syrian hamsters (A) **Lung images:** representative images of the lungs from mock-, rNDV-WT-, and rNDV-S-vaccinated hamsters at 2 and 4 DPI with SARS-CoV-2 are shown. Scale bars, 1 cm. (B) **Lung pathologic lesions:** macroscopic pathology scoring of lungs from hamsters in panel A was determined by measuring the distributions of pathological lesions (arrows), including consolidation, congestion, and pneumonic lesions using ImageJ software. (C and D) **SARS-CoV-2 titers:** SARS-CoV-2 titers in nasal turbinate (C) and lungs (D) from hamsters in panel A were determined by standard plaque assay. Dotted line indicates limit of detection (LOD, 200 PFU). Each symbol represents an individual animal, and @ represents one mock-vaccinated hamster that was removed because of accidental death and virus not detected in one mouse, #, virus not detected in two mice, ND, not detected. Lines represent the geometric mean. The Mann–Whitney test used for statistical analysis. ∗, P < 0.05; ∗∗, P < 0.01; or ∗∗∗, P < 0.005 for indicated comparisons.

To assess protection efficacy of rNDV-S against SARS-CoV-2 infection, hamsters were vaccinated (prime or boosted) with rNDVs (WT or S) and then challenged with 2 × 10^4^ PFU of SARS-CoV-2. For viral titration and pathology, hamsters were sacrificed at 2 and 4 days postinfection (DPI) (n = 4/group). Mock-vaccinated hamsters either challenged with SARS-CoV-2 or mock challenged were used as internal controls. Following challenge, lungs from mock-vaccinated hamsters showed mild-to-moderate, multifocal pneumonic lesions and congestions at 2 DPI and higher inflammation scores characterized by moderate to severe locally extensive to diffuse bronchopneumonia and foamy exudate in the trachea at 4 DPI (Figure 7A). Similar results were observed in hamsters vaccinated with rNDV-WT (primed or prime + boost). Conversely, hamsters vaccinated with rNDV-S (prime + boost) showed significantly lower inflammation scores compared with those of mock or rNDV-WT vaccinated hamsters at both 2 (P < 0.005) and 4 DPI (P < 0.05) (Figures 7A and 7B).

Viral titers from nasal turbinate and lung followed similar trends to the lung pathology and further support the lung inflammation scoring. Importantly, we were not able to detect the presence of SARS-CoV-2 in the nasal turbinate and lungs of hamsters vaccinated with rNDV-S (boosted). Hamsters vaccinated with a single dose of rNDV-S (prime) showed SARS-CoV-2 titers in nasal turbinate and lungs at 2 DPI similar to those of mock-vaccinated hamsters or hamsters vaccinated with rNDV-WT (Figures 7C and 7D). However, at 4 DPI, we could not detect the presence of SARS-CoV-2 in the nasal turbinate or lungs of hamsters vaccinated with rNDV-S, contrary to the situation of mock-vaccinated hamsters or hamsters vaccinated with rNDV-WT, where high titers (5.0 log10 PFU/ml) of SARS-CoV-2 were detected at 4 DPI (Figures 7C and 7D).

**Figure 8:**
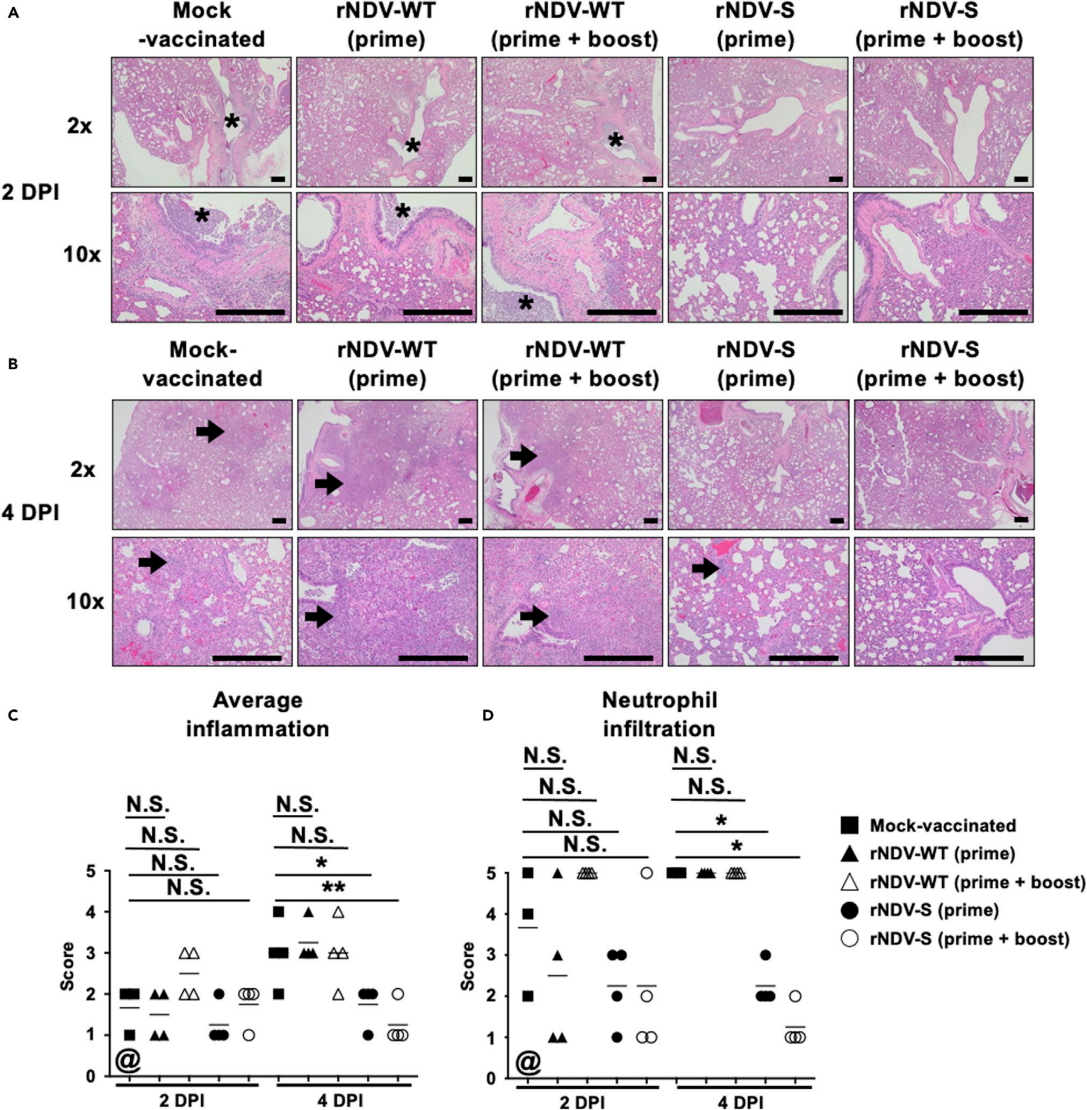
Histopathologic analysis of SARS-CoV-2 challenged golden Syrian hamsters (A and B) **HandE images of lungs from SARS-CoV-2-challenged hamsters at 2 DPI** (A) **and 4 DPI** (B)**:** lung lesions are primarily characterized by suppurative inflammation within and surrounding the bronchi/bronchiole (black asterisks) at 2 DPI and extending to bronchointerstitial inflammation (arrows) by 4 DPI. Scale bars, 500 μm. (C) **Average inflammation scoring:** the average inflammation scores were determined based on percent of inflamed lung area: grade 0 = no histopathological lesions, grade 1 = minimal (<10%) histopathological lesions, grade 2 = mild (10%–25%) histopathological lesions, grade 3 = moderate (25%–50%) histopathological lesions, grade 4 = marked (50%–75%) histopathological lesions, and grade 5 = severe (>75%) histopathological lesions. (D) **Neutrophil infiltration:** the neutrophil infiltration scores were graded base on lesion severity as follows: grade 0 = no lesions observed, grade 1 = <10 cells, grade 2 = <25 cells, grade 3 = <50 cells, grade 4 = >100 cells, and grade 5 = too many cells to count. The Mann–Whitney test was used for statistical analysis. ∗, P < 0.05; ∗∗, P < 0.01; or ∗∗∗, P < 0.005 for indicated comparisons. Lines represent the geometric mean. @ represents one mock-vaccinated hamster that was removed because of accidental death.

Histopathological analysis further confirmed these results (Figure 8). At 2 DPI, lungs from mock- or rNDV-WT-vaccinated hamsters showed abundant infiltration of inflammatory cells such as degenerate and nondegenerate neutrophils, macrophages, lymphocytes, plasma cells, and few eosinophils within the lumen of bronchi and bronchioles and surrounding small blood vessels (Figure 8A). At 4 DPI, histologic lesions were primarily characterized by extensive infiltration of the alveolar septa and alveolar space by neutrophils, macrophages and lesser lymphocytes, plasma cells, and few eosinophils. The bronchi and bronchioles showed a slightly lesser degree of inflammation with an average score of 1.8 at 2 DPI compared with 3.0 at 4 DPI in mock-vaccinated animals (Figure 8B). No significant differences in inflammation (Figure 8C) and neutrophil infiltration scores (Figure 8D) were seen between the different groups at 2 DPI. However, the degree of neutrophil infiltration and lung inflammation scores were significantly decreased in the rNDV-S-vaccinated groups (inflammation score of 2.25 for prime and of 1.25 for boosted) compared with that of mock-vaccinated (inflammation score of 5) or infected with rNDV-WT (inflammation scores of 5 for both prime and boosted) groups at 4 DPI (Figure 8D). Lungs of SARS-CoV-2-infected hamsters vaccinated with rNDV-S showed lower lung inflammation (score of 1.2) and numbers of neutrophil infiltration (score of 1.2) than those mock- (lung inflammation score 3.0 and neutrophil infiltration score of 5.0) or rNDV-WT-vaccinated (lung inflammation score 3.2 and neutrophil infiltration score of 5.0) hamsters, indicating that a booster vaccination with rNDV-S protects hamsters from SARS-CoV-2 infection and subsequent lung damage (Figure 8).

**Figure 9:**
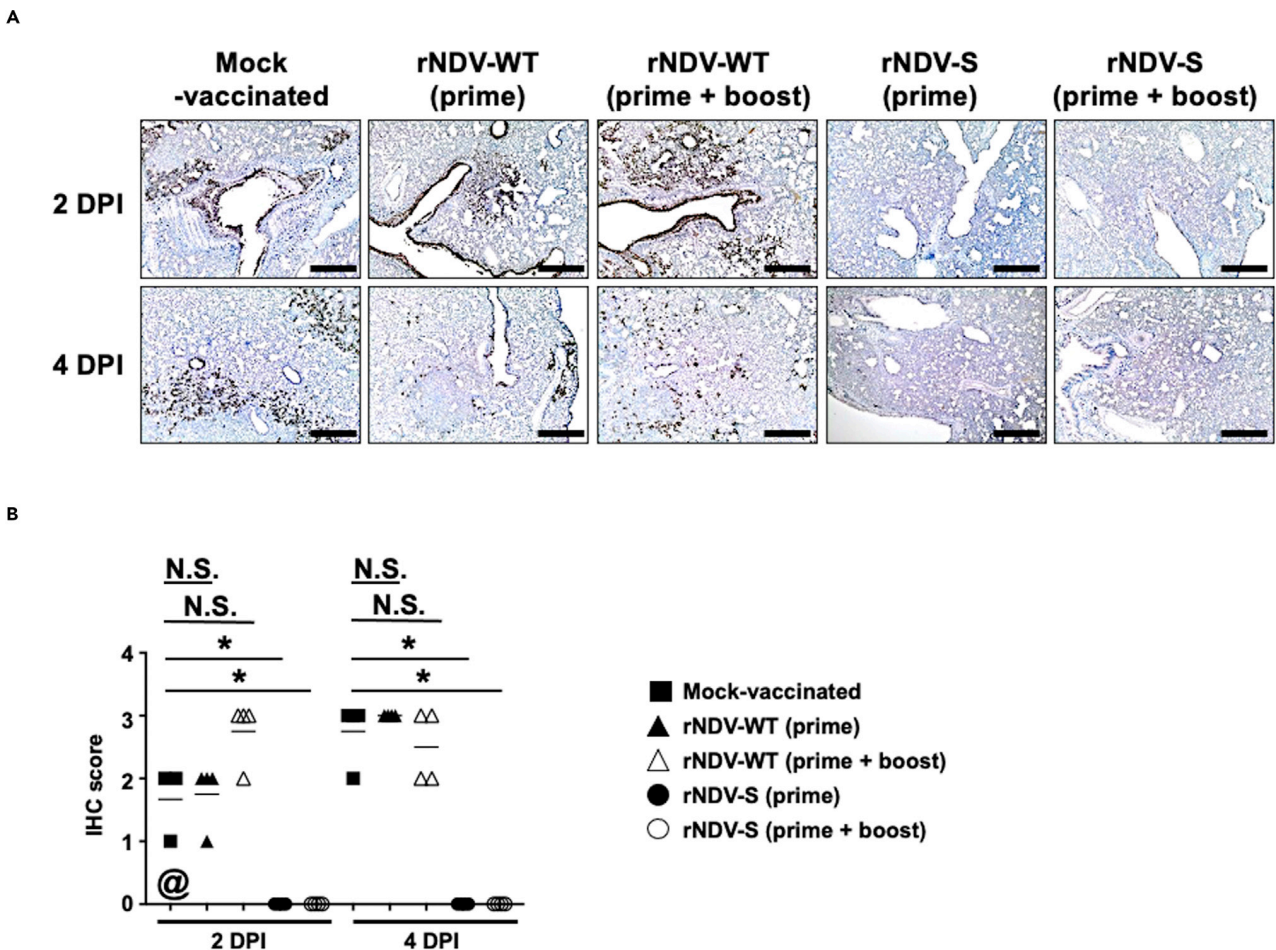
IHC analysis in SARS-CoV-2 challenged golden Syrian hamsters (A) **IHC imaging:** presence of SARS-CoV-2 viral N protein in the lungs of SARS-CoV-2-challenged hamsters at 2 and 4 DPI. Scale bars, 500 μm. (B) **IHC scores:** Scores were determined on the presence of SARS-CoV-2 N protein staining: grade 0 = no or rare immunostaining, grade 1 = viral N protein staining only in bronchi/bronchiole, grade 2 = viral N protein staining in bronchi/bronchiole and surrounding alveolar septa, grade 3 = viral N protein staining in alveolar septa distant from the bronchi and bronchioles, grade 4 = viral N protein staining throughout the lung. @ represents one mock-vaccinated hamster that was removed because of accidental death. The Mann–Whitney test was used for statistical analysis. ∗, P < 0.05; ∗∗, P < 0.01; or ∗∗∗, P < 0.005 for indicated comparisons.

Finally, IHC staining was performed to evaluate the presence and spread of SARS-CoV-2 replication by detecting viral N protein in the lungs from mock-, rNDV-WT-, or rNDV-S-vaccinated hamsters. As expected, SARS-CoV-2 N protein staining was not detected in any of the lungs from rNDV-S-vaccinated groups (prime or boosted) at 2 and 4 DPI, whereas lungs from mock- or rNDV-WT-vaccinated hamsters showed significant SARS-CoV-2 N protein positively stained cells. At 2 DPI, SARS-CoV-2 viral N protein staining was primarily within the epithelium of bronchi, bronchioles, and in inflammatory cells present in the lumen of bronchi and bronchioles and extended mild to rarely to the alveolar septa surrounding the bronchioles. At 4 DPI, SARS-CoV-2 N protein positive straining was present in the epithelium of bronchioles and along the edges of areas of bronchointerstitial inflammation within the inflammatory cells and alveolar septa that are distant from bronchioles (Figure 9A). The average IHC scores showed progressive and widespread distribution of SARS-CoV-2 in mock- and rNDV-WT-vaccinated groups and marked absence of viral N protein staining at 2 and 4 DPI in the rNDV-S-vaccinated groups (Figure 9B). These results demonstrate that vaccination with rNDV-S can protect from SARS-CoV-2 infection, resulting in lower SARS-CoV-2 titers and protection from lung damage.

## Discussion

Because of the high transmissibility of SARS-CoV-2 and lack of considerable preexisting immunity, the COVID-19 pandemic is posing significant health challenges, particularly in the elderly and those with existing comorbidities (Banerjee et al., 2020; Lee et al., 2020; Kang and Jung, 2020). Although both treatments and vaccines are being developed (Casadevall and Pirofski, 2020), there are limited attempts to offer vaccines that can be deployed in frail and resource-limited health care systems particularly in low- and middle-income countries. Upper- and middle-income countries have purchased the majority of currently available COVID-19 vaccines; global parity in vaccine access would undoubtedly induce both transmission and impact of the pandemic. In this regard, a low-cost vaccine production system for mass immunization of people in low- and middle-income countries is required. Here, we describe a promising and scalable live-attenuated vector vaccine based on the use of an rNDV expressing SARS-CoV-2 S, that can be administered intranasally to induce protection at the site of SARS-CoV-2 infection. This vaccine can be produced both robustly and economically in avian embryonated eggs, as well as in US FDA-approved cell lines.

Our studies establish that intranasal vaccination with rNDV-S induces Abs, including NAbs, against the full S protein of conventional and various variants (B.1.17, B.1.315, and P.1) of SARS-CoV-2, as well as T cell responses. Although a single dose conferred protection against SARS-CoV-2 challenge, a prime and boost regime provided superior protection, reduced lung pathology, and significantly reduced shedding of SARS-CoV-2 in hamsters, which are an appropriate animal model for vaccine evaluation (Sun et al., 2020a, 2020b; Tostanoski et al., 2020). Although SARS-CoV-2 replication was detected in nasal turbinate and lungs at 2dpi, animals cleared the challenge virus at 4dpi. Clearance of virus in the respiratory tract is plausible; however, it may merely be just below the detection limit of the assay and thus requires future transmission studies that are beyond the scope of this study. The intranasal route for immunization is advantageous in comparison to traditional routes (i.e., intramuscular) mainly due to its ability to elicit immunity at the local mucosal level (Ainai et al., 2013; Wang et al., 2015; Zheng et al., 2018). Enhancing mucosal protection is desirable, being the first line of defense in the human body against respiratory pathogens. Intranasal antigen delivery is associated with induction of mucosal immunity including production of IgA and priming of T and B cells in the nasopharynx-associated lymphoid tissues (Lycke, 2012). In addition, intranasal delivery may provide cross-protection by eliciting immunity at different mucosal sites such as the intestines and genital tract, which have been shown to be potential replication sites for SARS-CoV-2 (Cui et al., 2020; Zhang et al., 2020). Furthermore, intranasal delivery offers a needle-free route to vaccination as well as the potential for self-administration. It has previously been proposed that parenteral route of administration induced better protection over nasal administration (Harmsen et al., 2011), which may be dependent on the virus under investigation and nature of corelate of protection. We have observed weight loss within the first 4 days of intranasal vaccination, which could be attributed to the S protein expression by the NDV vector or route of administration. Although most of these animals recovered within the next 3 days, a comparative protective efficacy of rNDV-S using intramuscular versus intranasal routes is warranted in future studies. Although we observed no adverse pathology or any basic behavioral changes from our scoring system in the vaccine-alone treated mice and hamster models, we did detect a significant, albeit transient, early weight loss in both cases. Given this only occurred in the spike containing vaccine groups, it may indicate a stronger immune response and potential cytokine driven satiety or given the known gut expansion alterations to satiety driving hormones. Further examination of these early timepoints therefore would be of merit.

The correlation between SARS-CoV-2 viral load, viral manifestation, clinical symptoms, and the variety of immune cells associated with viral immunity are a topic of ongoing debate. However, the site of cellular infiltration and kinetics of cellular and humoral immunity are by far the most influential factors determining the outcome of the infection. CD8+ T cells are critical in the fight against viral infections, as their cytotoxic characteristics are designed to target intracellular threats such as viruses. CD4+ T cells on the other hand drive the immune milieu toward a Th1 immune response characterized by IFNγ-mediated immune responses and subsequent IgG2a Ab release. Current evidence indicates that Th1 type responses are key in the successful control of SARS-CoV-2 (Fara et al., 2020). In our study, spleen CD4+ T cell IFNγ+ levels increased systemically postvaccination in both prime and boost groups. Locally, in the lung tissue, increased IFNγ levels from both CD4+/CD8+ T cells were observed. This, in line with previously discussed data potentially provides a level of protective immunity in the form of IFNγ-antiviral functions (Tostanoski et al., 2020). Heightened TNF-α levels have been associated with disease severity. TNF-α and IL-10 directly correlate with COVID-19 severity and are synonymous with patient deterioration (Pedersen and Ho, 2020). In SARS-CoV-2 infection, TNF-α seems to lean toward detrimental rather than protective roles, especially as a key component of the group of cytokines known as the cytokine storm (Chen et al., 2020). In our work, a reduction in lung CD8+ T cell TNFα+ levels were associated with increased CD8+ T cell percentages; this potentially suggests that in our model, the vaccine promotes cytotoxic phenotype for CD8+ T cells independent of TNF-α.

Overall induction of IgM, IgA, and IgG as well as NAbs have been reported in numerous natural infections and in vaccine trials (Hassan et al., 2020). Coronavirus-specific IgM production is transient and leads to an isotype switch to IgA and IgG; these latter Ab subtypes can persist for extended periods in the serum and in nasal fluids (Park et al., 2021). After two doses of intranasal vaccination, we hypothesized the greater protection observed was because of the mucosal immune responses generated. In our study, at day 19 post immunization, local IgM levels were not increased in either FALC or plural fluid, suggesting a natural transition of the Ab phenotype toward an adaptive response from an innate immediate reaction.

Interestingly, studies have reported earlier peaks in anti-S protein IgA in mild cases earlier than peaks in IgM (Cervia et al., 2021). This increase suggests a more robust effect for IgA in COVID-19 in protection and clinical outcome of the disease (Yu et al., 2020). Indeed, high levels of anti-SARS-CoV-2 IgA were detected in serum and lung, and B cells secreting IgA were detected in the spleen only in mice vaccinated via an intranasal route. Moreover, intranasal, but not intramuscular, vaccination induced SARS-CoV-2-specific CD8+ T cells in the lung, including CD103+CD69+ cells, which are likely of a resident memory phenotype (Hassan et al., 2020). Future Ab passive transfer and T cell depletion studies can assess the relative contribution of each arm of the immune system and establish more precisely the mechanistic basis for the enhanced protection conferred by intranasal delivery of rNDV-S vaccine.

In our study, we noted an increase in specific anti-S RBD-IgA levels in the pleural cavity, and this corroborated with previous findings associating with mild COVID-19 infections where anti-S RBD-IgA levels were elevated at different mucosal tissues in recovered COVID-19 patients (Sterlin et al., 2021; Yu et al., 2020). Conversely, in our study, anti-S RBD-IgG2a production increased in the pleural cavity. This localized expansion of IgG2a, in particular, suggests a promotion of an Fc-mediated killing of the target cell through an antibody-dependent cellular cytotoxicity (ADCC) type of protection in response to the vaccine. In order to assess the relative contribution of each arm of the immune system to the conferred protection against SARS-CoV-2 via intranasal delivery of the rNDV-S vaccine, future studies are warranted.

Currently, there are limited SARS-CoV-2 vaccine platforms in clinical trials using an intranasal delivery method. Similar to influenza LAV, which induces profound local humoral and cellular responses (Calzas and Chevalier, 2019), immunization with rNDV-S offer superior properties over the intramuscular route. In addition, sterilizing immunity against reinfection or blockage of viral shedding requires local and mucosal immunity against SARS-CoV-2, which is optimally provided by the intranasal route (Laurie et al., 2010; Dutta et al., 2016). Recently, Sun et al. (2020a), (2020b) used NDV to express the ectodomain of the S gene without the cleavage site and utilized a murine model to demonstrate the protective efficacy of both killed and live vaccine against mouse adapted SARS-CoV-2 challenge (Sun et al., 2020a, 2020b). In these studies, mice were administered vaccines via the intramuscular route, and limited immunological investigations were undertaken. However, these studies outlined the potential use of NDV as a COVID-19 vaccine candidate. To further investigate the potential of NDV as a COVID-19 vaccine candidate, we used a pneumotropic NDV strain in a suitable hamster model challenged with wild-type SARS-CoV-2 and explored the immunological basis and intranasal potential of an NDV-based recombinant vaccine. Indeed, intranasal vaccination with rNDV-S induced the secretion of IgA and Nabs against conventional and variants (B.1.1.7, B1.315 and P.1) strains of SARS-CoV-2. It was also noticed that increasing the interval between prime and boost doses may enhance the memory response and increase humoral immunity. However, vector induced immunity may pose a complication in two doses vaccination regimen, which requires mitigation through non-NDV based vaccine administration. In addition, structure-guided design of antigen in prefusion (2P or HexaPro) can further enhance the immune induction, which warrants further study (Hsieh, et al., 2020). Nevertheless, the induction of immunity following administration of an intranasal vaccine does not require highly skilled personnel and also avoids the issues of individuals who experience trypanophobia and hemophobia.

In summary, our studies establish that intranasal immunization with rNDV-S induces both NAbs against SARS-CoV-2 variants (B.1.17, B.1.315 and P.1) and T cell responses in mice. Intranasal administration of two doses fully protected hamsters against lung infection, inflammation, and pathology following SARS-CoV-2 challenge. Importantly, a double dose of rNDV-S significantly reduces SARS-CoV-2 shedding in the nasal turbinate and lungs of hamsters. Thus, intranasal vaccination with rNDV-S has the potential to control SARS-CoV-2 infection at the site of inoculation, which should prevent both virus-induced COVID-19 disease and transmission. Based on these preclinical results in two rodent models, future clinical evaluation of rNDV-S in humans for the treatment of SARS-CoV-2 infection is urgently warranted.

### Limitations of the study

The intranasal vaccine candidate provides convincing efficacy against conventional strains of SARS-CoV-2, whereas the serum from vaccinated animals showed equivalent neutralization titers against B117 variant of SARS-CoV-2. However, the efficacy of the vaccine has not yet been established against recent mutants of SARS-CoV-2 such as P1 and B1.1351.

## STAR★Methods

### Key resources table

**Table:** 

REAGENT or RESOURCE	SOURCE	IDENTIFIER
**Antibodies**		

IgG1 HRP avidin-horseradish-peroxidase conjugate	Bio-Rad	101001
HRP-conjugated anti-hamster IgG	Jackson ImmunoResearch	AB_2338975
Anti-NP monoclonal Ab	A gift from Dr. Thomas Moran, USA	1C7
Anti-FcγR Ab	eBioscience and Biolegend	Cat# 14-0161-82, RRID:AB_467133
Anti-Ly6G	Biolegend	Cat# 127628, RRID:AB_2562567
Anti-Siglec F	BDBiosciences	Cat# 562681, RRID:AB_2722581 Cat# 562757, RRID:AB_2687994
Anti-CD19	Biolegend	Cat# 115538, RRID:AB_11203527
Anti-TCRβ	Biolegend	Cat# 109229, RRID:AB_10933263
Anti-CD11c	Biolegend	Cat# 117333, RRID:AB_11204262
Anti-CD45	Biolegend	Cat# 103151, RRID:AB_2565884
Anti-CD5	Biolegend	Cat# 100603, RRID:AB_312732
Anti-Ly6C	Invitrogen	Cat# 128007, RRID:AB_1186133
Anti-CD11b	Biolegend	Cat# 101255, RRID:AB_2563647
Anti-IA/IE	Biolegend	Cat# 107651, RRID:AB_2616728
Anti-CD3	Thermofisher Scientific	Cat# 56-0033-82, RRID:AB_837094
Anti-CD4	Thermofisher Scientific	Cat# 25-0041-82, RRID:AB_469576
Anti-CD49b	Thermofisher Scientific	Cat# 25-5971-82, RRID:AB_469667
Anti-IL-13	Thermofisher Scientific	Cat# 48-7133-82, RRID:AB_11219690
Anti-IFNγ	Thermofisher Scientific	Cat# 11-7311-41, RRID:AB_10718840
Anti-Ki67	Miltenyi Biotec	Cat# 130-111-929, RRID:AB_2652562
Anti-IL-17 (eBio17B7)	Thermofisher Scientific	Cat# 11-5773-82, RRID:AB_465243
Anti-Foxp3 (FJK-16s)	Thermofisher Scientific	Cat# 12-7321-82, RRID:AB_466199

Anti-TNFα	Thermofisher Scientific	Cat# 12-7321-82, RRID:AB_466199
Anti-IgA	Thermofisher Scientific	Cat# SA5-10308, RRID:AB_2868355
Anti-IgG2a	Thermofisher Scientific	Cat# 553391, RRID:AB_394829

**Bacterial and virus strains**		

SARS-CoV-2 USA-WA1/2020	BEI Resources	NR-52281
nCoV19 isolate/England ex-SA/HCM002/2021	European Virus Archive global	004V-04071
nCoV19 isolate/England/MIG457/2020	European Virus Archive global	004V-04032
2019 nCoV/Victoria/1/2020	Public Health England	NA

**Chemicals, peptides, and recombinant proteins**		

Recombinant SARS-CoV-2 spike protein	This paper	NA
Recombinant SARS-CoV-2 RBD protein	This paper	NA
Drosophila EX-CELL® 420 Serum-Free Medium	Merck Life Science	14420C
CaptureSelect™ C-tag Affinity Matrix	ThermoFisher Scientific	191307010
3,3',5,5'-tetramethylbenzidine (TMB) chromogen	Merck Life Science	T0440
NanoLuc Luciferase	Promega	N1110
DAB Peroxidase Substrate	Vector Laboratory	SK-4100
Collagenase D	Roche	COLLD-RO
MACH 4 Universal HRP Polymer	Biocare Medical	M4U534
Betazoid DAB Chromagen	Biocare Medical	BDB2004

Experimental models: Cell lines		
Cercopithecus aethiops: VeroE6	ATCC	CRL-1586
*Drosophila melanogaster*: Schneider 2 (S2)	ExpreS2ion Biotechnologies	NA
*Human*: 293T	ATCC	CRL-3216
Splenocyte and lungs primary cells	This paper	NA

**Experimental models: Organisms/strains**		

BALB/c female mice	Charles River, UK	NA
Syrian hamsters	Charles River, USA	NA

**Oligonucleotides**		

5’-AGTGATGTGCTCGGACCTTC-3	Wise et al. (2004)	NA
5’-FAM]TTCTCTAGCAGTGGGACAGCCTGC[TAMRA]-3’	Wise et al. (2004)	NA
5’-CCTGAGGAGAGGCATTTGCTA-3’	Wise et al. (2004)	NA

**Recombinant DNA**		

pCCNanoLuc2AEGFP	Schmidt et al., 2020	NA
pHIVNLGagPol	Schmidt et al., 2020	NA
pSARS-CoV-2-Strunc	Schmidt et al., 2020	NA
CS(ACE2)IB	Schmidt et al., 2020	NA

**Software and algorithms**		

FlowJo	TreeStar	https://www.flowjo.com/
Statistics: Prism 8.0	GraphPad	https://www.graphpad.com/scientific-software/prism/
Fiji	Image J	https://imagej.net/Fiji

### Resource availability

#### Lead contact

Further information and requests for resources and reagents should be directed to and will be fulfilled by the lead contact, Muhammad Munir, PhD (muhammad.munir@lancaster.ac.uk).

#### Materials availability

All requests for resources and reagents should be directed to and will be fulfilled by the Lead Contact, Muhammad Munir, PhD (muhammad.munir@lancaster.ac.uk). This includes selective antibodies, viruses, serum and proteins. All reagents will be made available on request after completion of a Materials Transfer Agreement.

### Additional information

Supplementary information is available for this paper.

### Experimental model and subject details

#### Ethics statement

All mice experiments were performed under the regulations of the Home Office Scientific Procedures Act (1986), specifically under the project licence PPL PACEB18AC. The project licence was approved by both the Home Office and the local AWERB committee of Lancaster University. All the *in vitro* and *in vivo* hamster experiments with infectious SARS-CoV-2 were conducted under appropriate BSL-3 and ABSL-3 laboratories, respectively, at Texas Biomedical Research Institute, San Antonio, Texas, US. Experiments were approved by the Texas Biomedical Research Institutional Biosafety (BSC) and Institutional Animal Care and Use (IACUC) committees, protocols BSC20-004.2 and 1722 MA 3, respectively.

#### Animals

BALB/c female mice were purchased from Charles River, UK and housed under specific pathogen free conditions at Lancaster University. Experiments were performed using age and sex matched mice at 11-14 weeks of age. Mice were provided sterilized water and chow *ad libitum* and acclimatized for minimum of one week prior to experimental manipulation. Specific-pathogen-free (SPF) 4-weeks-old female golden Syrian hamsters were purchased from Charles River, US. Hamsters were maintained in micro-isolator cages at Animal Biosafety Level (ABSL)-2 before and during vaccination with rNDV-WT or rNDV-S. For challenge with SARS-CoV-2, hamsters were transferred to ABSL-3. Hamsters were provided sterilize water and chow *ad libitum* and acclimatized for one week prior to experimental manipulation. All experiments in hamsters were conducted at Texas Biomedical Research Institute.

#### Cells and viruses

SARS-CoV-2, USA-WA1/2020 strain and B117 strain (GenBank: MN985325) were obtained from the Biodefense and Emerging Infections Research Resources Repository (BEI Resources, NR-52281) and amplified in African green monkey kidney epithelial Vero E6 cells obtained from the American Type Culture Collection (ATCC, CRL-1586) as previously described (Case et al., 2020). SARS-CoV-2 strains for B.1.315 and P.1 were obtained from European Virus Archive. Briefly, the virus stock obtained from original sources were regarded as passage four (P4) was amplified two more times to generate a P6 working stocks for animal infections and *in vitro* studies. To that end, Vero E6 cells were infected at low multiplicity of infection (MOI, 0.01) for 72 h and tissue culture supernatants (TCS) were collected, clarified, aliquoted and stored at -80°C until use. Virus stocks were titrated in Vero E6 cells by plaque assay and immunostaining as previously described (Mendoza et al., 2020; Park et al., 2021). The S gene of stock challenge viruses were sequenced and no changes in the length of the S gene including cleavage site was observed (Figure S10). The rNDV-WT and rNDV-S viruses were propagated in chicken embryonated eggs quantified in Vero cells using procedures we described before^33^. 293T cells and Vero cells were maintained in Dulbecco's Modified Eagle Medium (DMEM) with 10% FBS as described previously (Rohaim and Munir, 2020).

### Method details

#### Safety and immunogenicity studies in mice

Mice were assigned to 4 experimental groups receiving a 50 μl intranasal dose of either mock (PBS) vehicle at days 0 and 14; 10^4^ PFU of rNDV-WT at days 0 and 14 (booster) or rNDV-S at day 0 only (prime) or at days 0 and 14 (booster). Mice and chow were weighed daily and nasally swabbed at times indicated before sacrifice at 19 DPV. Mouse morbidity was scored using a 14 point scoring system based on a) Hunched posture: Yes (1), No (0); b) Spontaneous activity: None (2), Reduced (1), Normal (0); c) Response to touch: None (3), Movement (2), Move away (1), Normal (0); d) Feels cold: Yes (1), No (0); e) Breathing laboured: Yes (1), No (0); f) “Rasping” breathing: Yes (1), No (0); g) Ruffled fur/piloerection: Yes (1), No (0); h) Pallor at extremities: Yes (1), No (0); and i) Moderate staining around eyes and/or nose.

#### Vaccine safety studies in hamsters

To evaluate *in vivo* safety of rNDV-S, hamsters (n=8 per group) were vaccinated intranasally with 1 x 10^6^ PFU of rNDV-WT or rNDV-S, or mock-vaccinated with saline (PBS), in a final volume of 100 μl, after sedation in an isoflurane chamber. Hamsters were vaccinated following a prime or a booster regimen. In the case of the booster immunization, animals were boosted 2 weeks after prime vaccination. After vaccination, hamsters were monitored daily for morbidity (body weight changes and clinical signs of infection) and mortality (survival) for 14 (prime) or 28 (booster) DPV. Sera were collected at 0 and 14 (mock, prime, and booster groups), and 28 (booster) DPV. One mock-vaccinated hamster in the vaccination group was removed because of accidental death.

#### Vaccine efficacy in hamster

After 14 (prime) or 28 (mock and booster groups) DPV, hamsters were challenged intranasally with 2 x 10^4^ PFU of SARS-CoV-2 in a final volume of 100 μl under isoflurane sedation. To evaluate SARS-CoV-2 titers in nasal turbinates and lung, hamsters were humanely sacrificed at 2 (n=4) or 4 (n=4) DPI. Nasal turbinates and lungs were harvested, and half of the organs were homogenized in 2 mL of PBS using a Precellys tissue homogenizer (Bertin Instruments) for viral titration and the other half was kept in 10% neutral buffered formalin (NBF, ThermoFisher Scientific) for histopathology and immunohistochemistry (IHC). Tissue homogenates were centrifuged at 21,500 x *g* for 5 min and supernatants were used to calculate viral titers.

#### Real-time PCR for quantification of rNDV replication

RNA was extracted from mice organs (nasal turbinate, lungs, trachea, and gut) using TRIzol™ reagent as per manufacturer’s instructions (Invitrogen, USA). Real-time qRT-PCR was performed using SuperScript™ III Platinum™ One-Step qRT-PCR Kit (Invitrogen, USA) following the manufacturer’s instructions to detect NDV M gene (Wise et al., 2004) and enable the calculation of NDV genome copies.

#### Production and purification of recombinant SARS-CoV-2 S and RBD proteins

Recombinant S and RBD proteins were produced and purified as previously described (Lukosaityte et al., 2020). Briefly, expression cassettes containing SARS-CoV-2 S and RBD nucleotide sequences were codon optimized for Drosophila melanogaster Schneider 2 (S2) cells. The N-terminus signal sequence of both S and RBD proteins were replaced with Drosophila BiP signal sequence which encodes immunoglobulin-binding chaperone protein, and the C-terminus were fused with T4 foldon sequence (GSG YIP EAP RDG QAY VRK DGE WVL LST FL) and a C-tag sequence (EPEA) for affinity purification. The protein expression cassettes were commercially synthesised (GeneArt, ThermoFisher Scientific, Regensburg, Germany) and cloned into pExpres2.1 expression vector (ExpreS2ion Biotechnologies, Hørsholm, Denmark). The recombinant plasmids were transfected into Drosophila S2 cells using Calcium Phosphate Transfection Kit (Thermo Fisher Scientific, Paisley, Scotland, UK). Following antibiotic selection with Zeocin (InvivoGen, Toulouse, France), cells were propagated in Drosophila EX-CELL® 420 Serum-Free Medium (Merck Life Science) at 25°C. Recombinant S and RBD trimeric proteins secreted in cell culture supernatants were purified using the CaptureSelect™ C-tag Affinity Matrix (ThermoFisher Scientific). Concentration of purified recombinant Abs were determined by PierceTM BCA Protein Assay Kit (ThermoFisher Scientific) and the purity was assessed by sodium dodecyl sulphate–polyacrylamide gel electrophoresis (SDS-PAGE).

#### Indirect antigen capture enzyme-linked immunosorbent assays (ELISA) for Ab detection in mice

SARS-CoV-2 S or RBD proteins were coated onto 96-well ELISA plates. Viral antigen proteins were diluted in PBS to a concentration of 5 μg/ml, using 100 μl per well. Following an overnight incubation at 4°C, plates were washed 3 times with PBS-Tween-20 (PBST) and blocked with 2% bovine serum albumin (BSA) in PBS overnight at 4°C. Sera samples were heat inactivated at 56°C for 30 min prior to testing by ELISA. Hundred μl of the diluted (1:10 in 2% BSA) primary Abs were added to each well and incubated for 3 h at 37°C. Following 3 times washing with PBST, plates were incubated with 1:6,000 IgG1 HRP avidin-horseradish-peroxidase conjugate diluted in 2% BSA for 2 h at 37°C. Following 3 times washing in PBST, colour was developed using 3,3',5,5'-tetramethylbenzidine (TMB) chromogen (100 μl per well) for 5 min at room temperature. The reaction was stopped with an equal volume of 0.16M H_2_SO_4_ and optical density (OD) was read at 450 nm. Sample to positive ratio (S/P), as defined by the formula S/P = (sample OD - standard negative OD)/ (standard positive OD - standard negative OD), were used for reporting all ELISA values.

#### ELISA for Ab detection in hamsters

Binding affinity of hamster sera to SARS-CoV-2 antigens was determined by ELISA using lysates from mock-vaccinated and SARS-CoV-2-infected Vero E6 cells. Briefly, ELISA plates were coated overnight at 4°C with a 1:1,000 dilution of cells extracts from mock- and SARS-CoV-2-infected Vero E6 cells. Plates were washed 3X with PBS and incubated with 2-fold, serially diluted hamster serum samples in PBS (starting dilution of 1:100) for 1 h at 37°C. Plates were washed 3X with PBS and incubated with HRP-conjugated anti-hamster IgG (Jackson ImmunoResearch, PA, USA) for 1 h at 37°C. After 3X washes with PBS, plates were developed with TMB substrate buffer. The reaction was stopped by 2M H_2_SO_4_ after incubation for 10 min at room temperature. The optical density (OD) values were measured at 450 nm using an ELISA plate reader.

#### Neutralization assays using pseudotyped viruses

Derivatives of 293T cells expressing ACE2 receptors were generated by transducing 293T cells with ACE2 receptor expressing vector. Human 293T cells expressing ACE2 were used for rescue of SARS-COV-2 S-pseudotyped HIV particles for neutralisation assays. To generate SARS-COV-2 S-pseudotyped HIV, the reporter vector (pCCNanoLuc2AEGFP), HIV-1 structural/regulatory proteins (pHIVNLGagPol) and pSARS-CoV-2-Strunc were used at a molar plasmid ratio of 1:1:0.45 and the infectivity of pseudotyped viral particles was calculated as described previously^34^. For neutralization assays of mice sera, sera (1:10 starting dilution) were five-fold serially diluted in 96-well plates. Thereafter, a 50 μl aliquot of HIV-based SARS-CoV-2 pseudovirus containing approximately 1×10^3^ infectious units were added. After 1h incubation at 37°C, 100 μl of the mixture was transferred to target cells plated at 1×10^4^ cells/well in 100 μl medium in 96-well plates. Cells were then cultured for 48h and cells were harvested for NanoLuc luciferase assays. For the NanoLuc luciferase assays, cells were washed twice, carefully, with PBS and lysed with 50 μl/well of Luciferase Cell Culture Lysis reagent (Promega). NanoLuc Luciferase activity in lysates was measured using the Nano-Glo Luciferase Assay System (Promega). Specifically, 25 μl of substrate in NanoGlo buffer was mixed with 25 μl cell lysate in black flat bottom plates and incubated for 5 min at RT. NanoLuc luciferase activity was measured using Glowmax Navigator luminometer (Promega), using 0.1s integration time. Relative luminescence units (RLU) obtained were normalized to those derived from cells infected with SARS-CoV-2 pseudovirus in the absence of Abs. The half maximal neutralizing concentration (NC_50_) for sera was determined using 4-parameter nonlinear regression curve fit to raw infectivity data measured as relative light units, or as the percentage of infected cells (GraphPad Prism).

#### SARS-CoV-2 microneutralization assay

The day before infection, Vero-E6 cells were seeded at 1×10^4^ cells/well into 96-well plates and incubated at 37°C in a 5% CO_2_ incubator overnight. Mice sera samples were heat inactivated at 56°C for 30 min. Sera samples were 10-fold serially diluted in DMEM (Invitrogen, USA) supplemented with 1% BSA and 1x penicillin/streptomycin. Next, the diluted sera samples were mixed with a constant amount of SARS-CoV-2 (100 Median Tissue Culture Infectious Dose (TCID_50_)/50 μl) and incubated for 1 h at 37°C. The Ab-virus-mix was then directly transferred to Vero E6 cells and incubated for 3 days at 37°C in a 5% CO_2_ incubator. The median microneutralization (MN) 50 (MN_50_) or 100 (MN_100_) of each serum sample was calculated as the highest serum dilution that completely protect the cells from cytopathic effect (CPE) in half or all wells, respectively.

#### Plaque reduction microneutralization (PRMN) assays

PRMN assays were performed to identify the levels of SARS-CoV-2 NAbs as described previously^30^. Briefly, sera samples were inactivated at 56°C for 30 min prior to assay. For pre-treatment conditions, 200 PFU/well of SARS-CoV-2 were mixed with 2-fold serial dilutions (starting dilution 1:100) of hamsters’ sera and incubated at 37°C for 1 h. Confluent monolayers of Vero E6 cells (96-well plate format, 4 x 10^4^ cells/well, quadruplicates) were infected with the virus-serum mixture for 1 h 37°C. After viral absorption, infectious media was exchanged with post-infection media containing 2% FBS and 1% Avicel. For post-treatment conditions, confluent monolayers of Vero E6 cells (96-well plate format, 4 x 10^4^ cells/well, quadruplicates) were infected with 100 PFU/well of SARS-CoV-2 for 1 h at 37°C. After viral adsorption, infection media was exchanged with 100 μl of post-infection media containing 2% FBS, 1% Avicel, and 2-fold serial dilutions (starting dilution 1:100) of hamster sera. In both cases (pre- and post-treatment conditions) infected cells were fixed 24 h post-infection (hpi) with 10% neutral formalin for 24 h and immunostained with a monoclonal Ab (1C7) against the viral nucleocapsid (N) protein (1 μg/ml). Viral neutralization was evaluated and quantified using ELISPOT, and a sigmoidal dose-response, non-linear regression curve was generated using GraphPad Prism to calculate median neutralization titer (NT_50_) in each of the serum samples.

#### SARS-CoV-2 virus titrations

Nasal turbinate and lungs from SARS-CoV-2-infected golden Syrian hamsters were homogenized in 2 ml of PBS for 20 s at 7,000 rpm using a Precellys tissue homogenizer (Bertin Instruments). Confluent monolayers of Vero E6 cells (96-plate format, 4 x 10^4^ cells/well, duplicates) were infected with 10-fold serial dilutions of supernatants obtained from the nasal turbinate or lung homogenates from SARS-CoV-2 infected golden Syrian hamsters. Virus from serially diluted samples was adsorbed at 37°C for 1 h followed by incubation in post-infection media containing 2% FBS and 1% Avicel at 37°C for 24 h. After viral infection, plates were submerged in 10% NBF for 24 h for fixation/inactivation. For immunostaining, cells were washed 3X with PBS and permeabilised with 0.5% Triton X-100 for 10 min at room temperature, followed by blocking with 2.5% bovine serum albumin (BSA) in PBS for 1 h at 37°C. Cells were incubated with the N protein 1C7 monoclonal Ab (1 μg/ml) diluted in 1% BSA for 1 h at 37°C. Then cells were washed 3X with PBS and stained with the Vectastain ABC kit and developed using the DAB Peroxidase Substrate kit (Vector Laboratory, Inc, CA, USA), as previously described (Case et al., 2020). Virus titers were calculated as PFU/ml.

#### Cell re-stimulation and flow cytometry staining

Spleens were removed from mice and disaggregated through a 100 μm sieve. Pericardium was digested using 1mg/ml Collagenase D (Roche) in a shaking heat block at 37°C, digestion was stopped after 35mins by addition of 5mM EDTA (Fisher) followed by passing through a 100μm cell strainer. Pleural exudate cells (PLEC) were isolated via lavage of the pleural cavity with 10ml of ice cold dPBS (Sigma). Left lung lobes were excised and finely diced using scissors prior to digestion in 2 ml lung digest medium (PBS (Sigma), 0.1mg/ml Liberase TM (Roche), 50 μg/ml DNAse I (Roche)) for 30 min in a shaking incubator at 37°C. Digestion was stopped after 30mins by adding 5mM EDTA (Fisher) followed by passing through a 100 μm cell strainer. Lung & spleen cells were diluted to 1x10^6^cells/ml before incubating with 50 μg/ml SARS-CoV-2 purified spike antigen for 24 h in media (RPMI-1640, 10% FCS, 100U/ml penicillin/streptomycin, 5%NEAA, L-glutamine and HEPES, 0.05 mM β-mercaptoethanol (SIGMA)). Cell suspensions were incubated for the final 6 h with 1x Cell stimulation cocktail (plus protein inhibitors) (eBioscience). Cell suspensions were blocked with anti-FcγR Ab (clone 24G2; eBioscience and Biolegend) before labelling with combinations of Abs specific for Ly6G (1A8; Biolegend), Siglec F (E50-2440; BD), CD19 (6D5, Biolegend), TCRβ (H57-597, Biolegend), CD11c (N418; Biolegend), CD45 (30-F11; Biolegend), CD5 (53-7.3; Biolegend), Ly6C (HK1.4; Invitrogen), CD11b (M1/70; Biolegend), IA/IE (M5/114.15.2, Biolegend), F4/80 (BM8, Invitrogen), CD3 (eBio500A2), CD4 (clone GK1.5; eBioscience, RM4-5, Biolegend), CD49b (DX5; EBioscience), IL-13 (clone eBiol13A; eBioscience), IFNγ (clone XMG1.2; eBioscience), Ki67 (REA183, Miltenyi) and IL-17(eBio17B7; eBioscience). For intracellular cytokine analysis cells were then stained with Abs using the eBioscience Foxp3 permeabilization kit according to the manufacturer's instructions. All samples were analysed on a Beckman Cytoflex and analysed with FlowJo Software (TreeStar).

#### Pathologic observation

Mouse lung tissues were inflated and fixed in 10% neutral buffered formalin (NBF) solution and embedded in paraffin prior to H&E. After mounting, positive cells were enumerated in field of view and alveolar space enumerated via NIH Image J software.

In the hamster studies, lungs were collected at 2- and 4- DPI from mock- and SARS-CoV-2-challenged golden Syrian hamsters. Lung samples were photographed to show gross lesions on both the dorsal and ventral views. Images were used for macroscopic pathology scoring analysis by measuring the distributions of pathological lesions, including consolidation, congestion, and pneumonic lesions using NIH ImageJ software. The area of pathological lesions was converted into percent of the total lung surface area. Half of the hamster lungs fixed with 10% neutral buffer formalin (NBF) were embedded in paraffin blocks, and sectioned (5 μm). Sections were stained with H&E and evaluated under light microscopy in a blinded manner by a board-certified veterinary pathologist at Texas Biomedical Research Institute. The average inflammation scores were graded based on percent of inflamed lung area: grade 0 = no histopathological lesions observed, grade 1 = minimal (< 10%), grade 2 = mild (10 to 25%), grade 3 = moderate (25 to 50%), grade 4 = marked (50 to 75%), and grade 5 = severe (> 75%). And the neutrophil infiltration scores were graded base on lesion severity as follows: grade 0 = no lesions observed, grade 1 = <10 cells, grade 2 = <25 cells, grade 3 = <50 cells, grade 4 = >100 cells, and grade 5 = Too numerous to count (TNTC).

#### Immunohistochemistry (IHC) assays

Immunostainings were performed as previously described (Chen et al., 2020). Briefly, 5 um lung tissues sections were mounted on Superfrost Plus Microscope slides, deparaffinized and antigen retrieval was conducted using the HIER method (Heat Induced Epitope Retrieval). Subsequently, slides were stained with the primary N protein monoclonal Ab 1C7 (1 μg/ml). Then slides were incubated and developed with the MACH 4 Universal HRP Polymer Kit and Betazoid DAB Chromagen Kit (Biocare Medical, LLC), according to the manufacturer’s instructions. The presence of SARS-CoV-2 N protein antigen was analysed by a veterinary pathologist at Texas Biomedical Research Institute. Scores of antigens were graded based on the anatomical location of viral N protein staining: grade 0 = no or rare immunostaining, grade 1 = viral N protein staining only in bronchi/bronchiole, grade 2 = viral N protein staining only in bronchi/bronchiole and surrounding alveolar septa, grade 3 = viral N protein staining in alveolar septa distant from the bronchi and bronchioles, grade 4 = viral N protein staining throughout the lung. The Mann–Whitney test was used for statistical analysis.

#### Confocal imaging

Mouse mediastinum samples were fixed for one hour on ice in 10% NBF (Sigma) and then permeabilised in PBS 1% Triton-X 100 (Sigma) for 15 min at room temperature prior to staining with primary Abs for one hour at room temperature in PBS 0.5% BSA 0.5% Triton. DAPI was added for the final 10 min of the 1 h incubation. After washing in PBS, tissues were mounted in fluoromount G and confocal images were acquired using a Zeiss LSM880 confocal laser scanning microscope. Samples were analysed using Fiji software. Cluster area was delineated using IgM, and percentage area of Ki67 & CD4 staining within each cluster was calculated.

### Quantification and statistical analysis

Results are expressed as mean ± SEM. Where statistics are quoted, two experimental groups were compared via the Student’s t test or the Mann–Whitney for non-parametric data. Three or more groups were compared with ANOVA, with Sidaks, Dunnett’s or Bonferroni’s post-test as indicated. A p value of <0.05 was considered statistically significant. ∗, P < 0.05; ∗∗, P < 0.01; or ∗∗∗, P < 0.005 for indicated comparisons, error bars represent standard error of means.

## Data Availability

This study did not generate/analyse any datasets/code.

## Footnotes

Supplemental information can be found online at https://doi.org/10.1016/j.isci.2021.102941.
