# Supplementary material for: Immunogenicity and protective efficacy of an intranasal live-attenuated vaccine against SARS-CoV-2
Source: iScience. 2021 Aug 4;24(9):102941. doi: 10.1016/j.isci.2021.102941 (PMC8332743; doi:10.1016/j.isci.2021.102941)
Supplement: Document S1. Figures S1–S10 and Table S1 [file mmc1.pdf]

iScience, Volume *24*

Supplemental information

Immunogenicity and protective efficacy of an intranasal live-attenuated vaccine against SARS-CoV-2

Jun-Gyu Park, Fatai S. Oladunni, Mohammed A. Rohaim, Jayde Whittingham-Dowd, James Tollitt, Matthew D.J. Hodges, Nadin Fathallah, Muhsref Bakri Assas, Wafaa Alhazmi, Abdullah Almilaibary, Munir Iqbal, Pengxiang Chang, Renee Escalona, Vinay Shivanna, Jordi B. Torrelles, John J. Worthington, Lucy H. Jackson-Jones, Luis Martinez-Sobrido, and Muhammad Munir

Supplementary Information

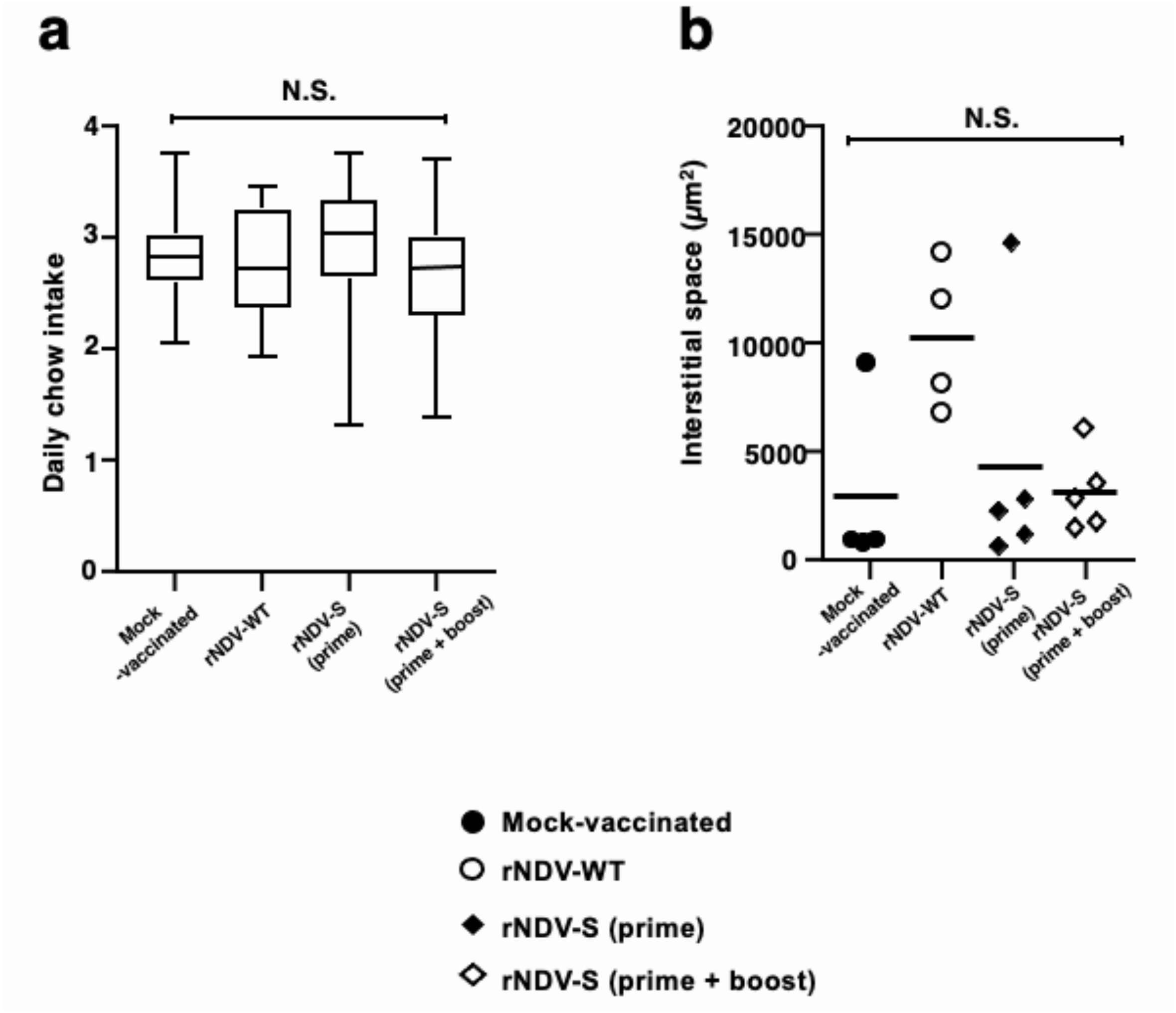

Supplementary Figure 1. Impact of rNDV-S intranasal vaccination on lung and chow intake, Related to Figure 1 (**a**) Daily chow intake by mock-vaccinated or rNDV-S vaccinated mice. (**b**) Mean interstitial space size of right lung following H&E staining and assessment via ImageJ. Data (n=4–5 mice/group), NS, non-significant between naïve and vaccinated groups, error bars represent SE of means via repeated t-test or ANOVA with Dunnett's post-test.

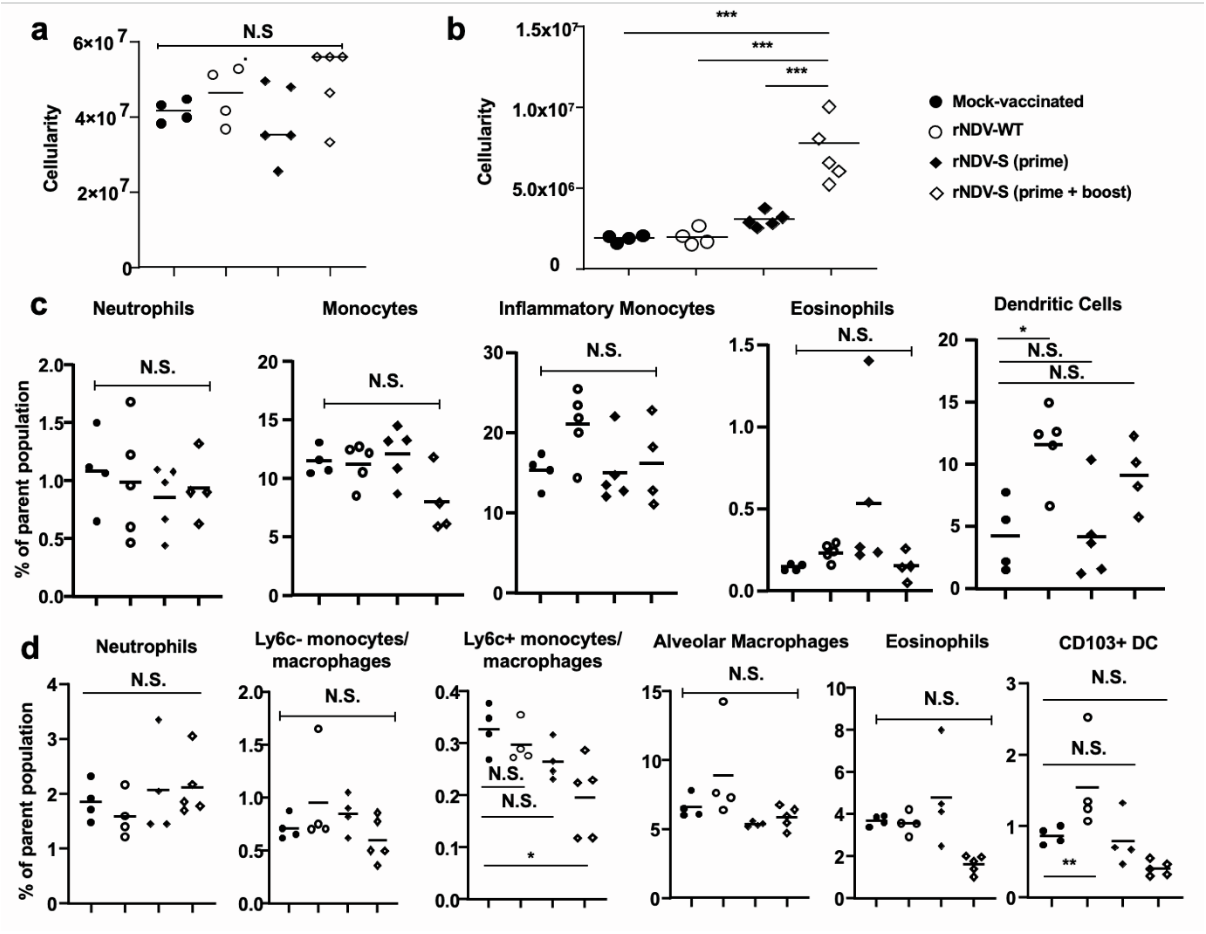

Supplementary Figure 2. No adverse myeloid inflammatory response within the spleen or lung following vaccination with rNDV-S, Related to Figure 1. Cellularity of spleen (**a**), lung (**b**) and flow cytometric analysis of splenic (**c**) and lung (**d**) myeloid cell populations from mice on day 19 following instillation of mock PBS or indicated rNDV constructs intranasally on days 0 & 7. Data (n=4–5 mice/group); *, P<0.05; **, P<0.01; or ***, P<0.005 between naïve and infected groups, error bars represent SE of means via ANOVA with Dunnett's post-test.

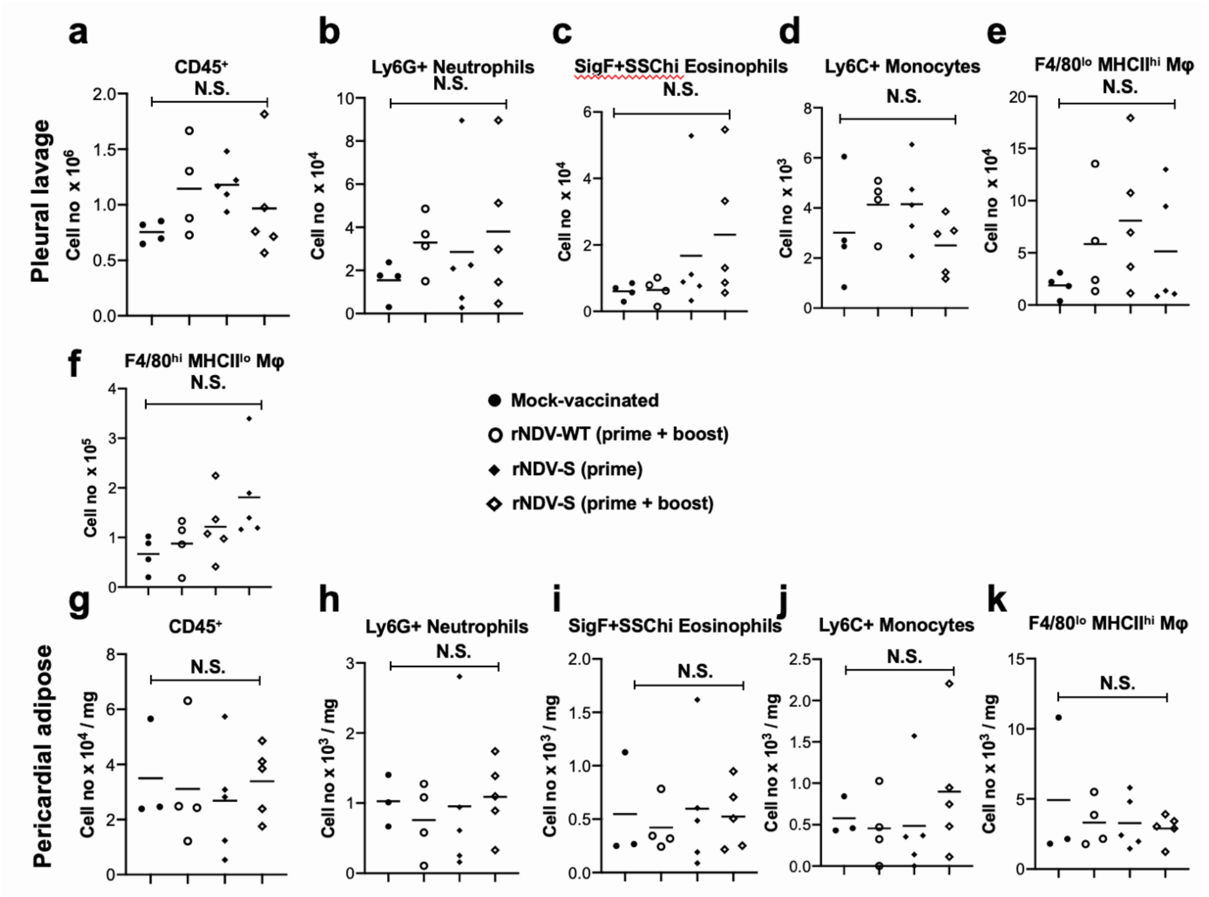

Supplementary Figure 3. No lasting inflammatory response within the pleural and pericardial cavities following vaccination with rNDV-S, Related to Figure 1. Flow cytometric analysis of pleural lavage (upper panels) and digested total pericardial adipose (lower panels) cells from mice on day 19 following instillation of PBS or indicated rNDV constructs, on day 0 & 7. In Pleural lavage, total number of, CD45^{+} cells **a)** Ly6G^{+} Neutrophils (**b**) SigF^{+}SSC^{hi} Eosinophils (**c**), Ly6C+ monocytes (**d**), F480^{lo}MHC-II^{hi} Macrophages (**e**) and F4/80^{hi}MHC-II^{lo} macrophages (**f**). In pericardial adipose, total number of, CD45^{+} cells **g)** Ly6G^{+} Neutrophils (**h**) SigF^{+}SSC^{hi} Eosinophils (**i**), Ly6C+ monocytes (**j**) and F480^{lo}MHC-II^{hi} Macrophages (**k**)

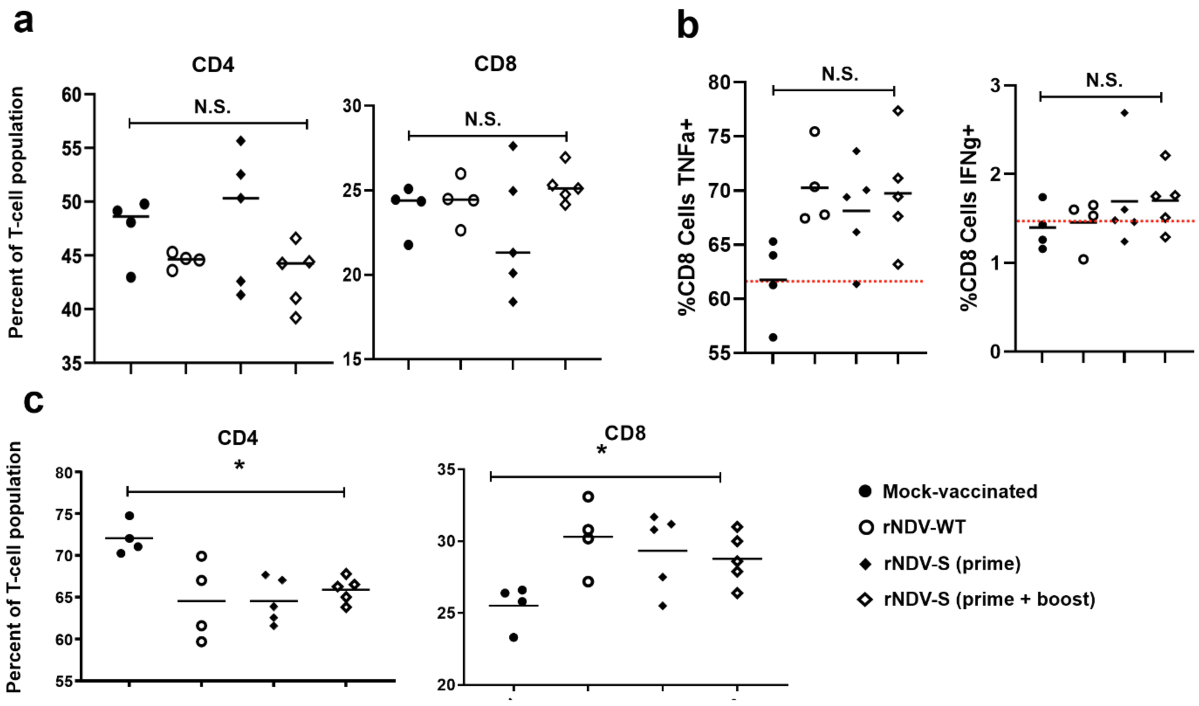

Supplementary Figure 4. T-cell population dynamics and splenic T-cell cytokine production following vaccination, Related to Figure 3. Flow cytometric analysis of splenic T-cell populations (**a**), cytokine responses to SARS-CoV-2 spike protein (**b**) and (**c**) lung T-cell populations from mice on day 19 following instillation of mock PBS or indicated rNDVs constructs intranasally on days 0 & 7. Red dashed line indicates baseline cytokine response to PMA/ionomycin without antigen. Data (n=4–5 mice/group); *, P<0.05; **, P<0.01; or ***, P<0.005 between naïve and infected groups, error bars represent SE of means via ANOVA with Dunnett's post-test.

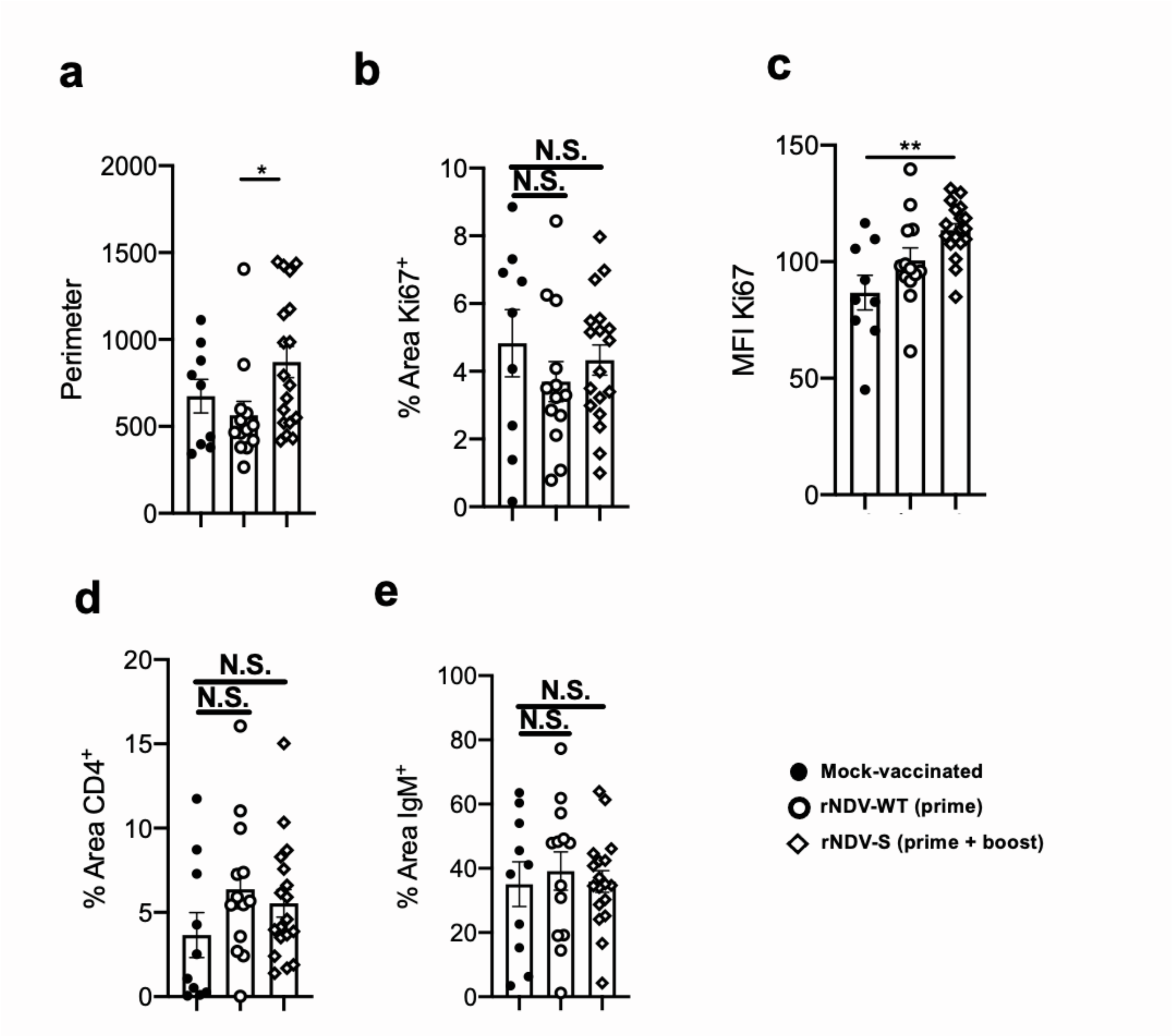

Supplementary Figure 5. Quantitative analysis of the fat associated lymphoid clusters (FALCs) expansion, Related to Figure 4. Whole mount immuno-fluorescence imaging of the murine mediastinum on day 19 following instillation of PBS or indicated rNDV constructs, intranasally, on days 0 and 7. Quantitative presentation of (**a**) cluster perimeter. (**b**) %Ki67^{+} area of cluster and (**c**) mean fluorescence intensity of Ki67 expression. (**d**) %CD4^{+} area of cluster. (**e**) %IgM^{+} area of cluster. Scale bar = 50μm.

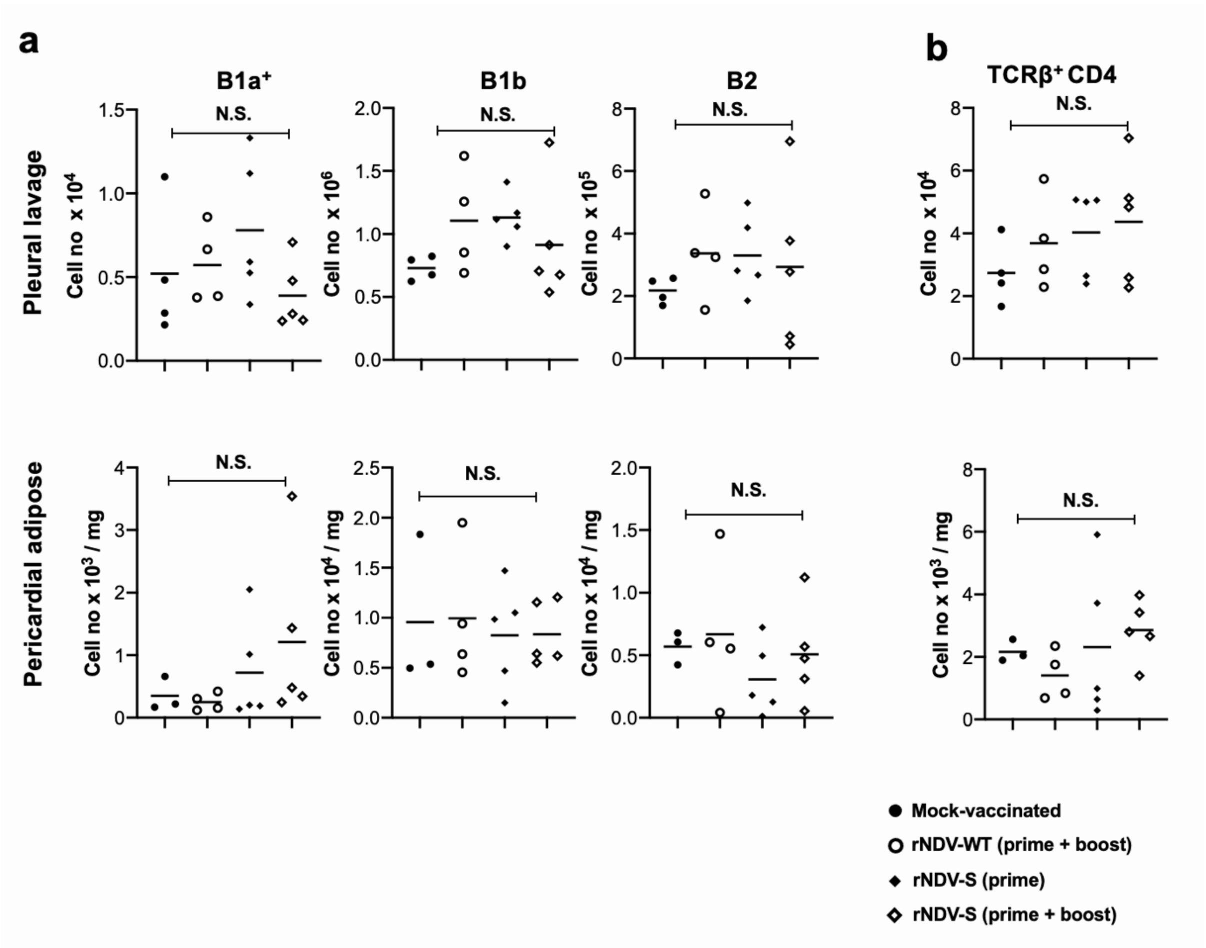

Supplementary Figure 6. **Adaptive immune cell populations within the pleural lavage and pericardial adipose, Related to Figure 4. (a)** Total $CD19^{+}MHCII^{+}CD11b^{+}CD5^{+}$ B1a, $CD19^{+}MHCII^{+}CD11b^{+}CD5^{-}$ B1b & $CD19^{+}MHCII^{+}CD11b^{-}$ B2 cells, (**b)** TCR**β**$^{+}$CD4^{+} T cells determined by flow cytometric analysis of pleural lavage (upper panels) and digested total pericardial adipose (lower panels) cells from mice on day 19 following instillation of PBS or indicated rNDV constructs intranasally on days 0 & 7.

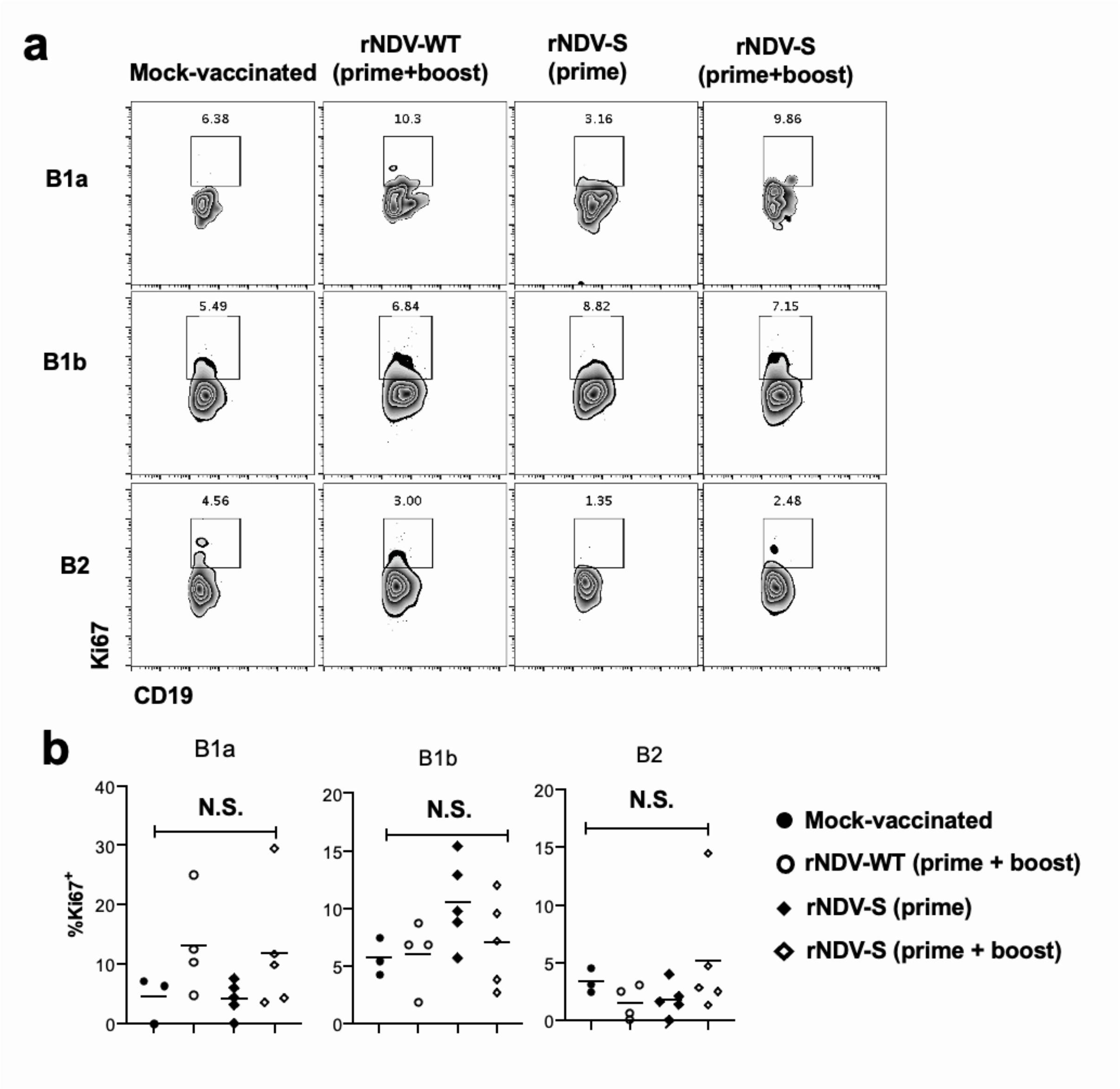

Supplementary Figure 7. Related to Figure 4. (**a**). Proliferation of pericardial B-cells isolated following digestion as determined via flow cytometric analysis on day 19 following instillation of PBS or indicated rNDV constructs intranasally on days 0 & 7. **(b**) Quantitative presentation of different cell population shown in panel (**a**).

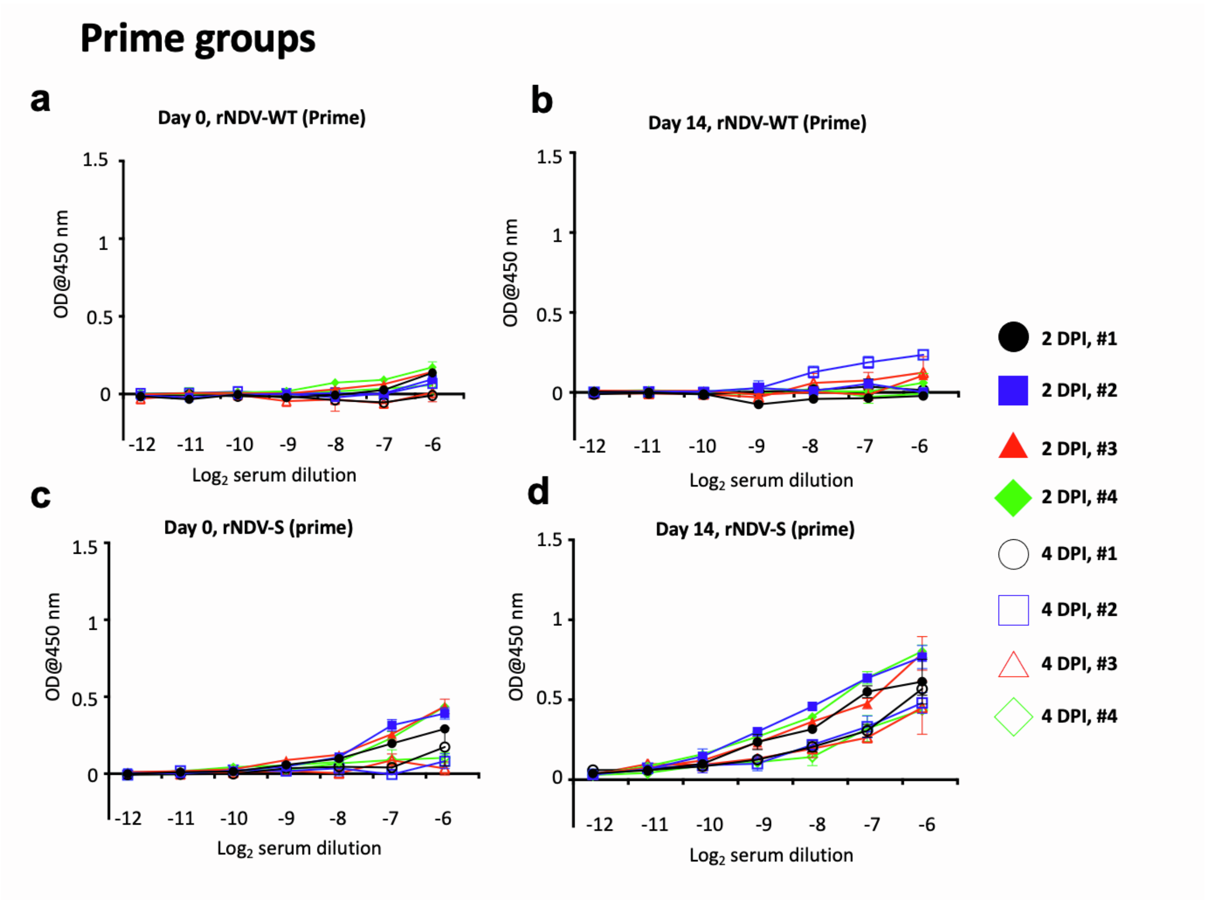

Supplementary Figure 8. Total Abs in serum of individual golden Syrian hamsters vaccinated with rNDV constructs, Related to Figure 6. Levels of total S binding Abs in sera from individual hamsters vaccinated with either rNDV-WT or rNDV-S at 0 DPV (prime). Sera from rNDV-WT infected animals were collected at 0 (**a**) and 14 DPV (**b**). Sera from rNDV-rNDV-S infected animals were collected at 0 (**c**) and 14 DPV (**d**). Individual hamster titres of Abs are displayed after 2 DPI and 4 DPI. The average of Abs titre in each group were presented in the Figure 6 in the main manuscript.

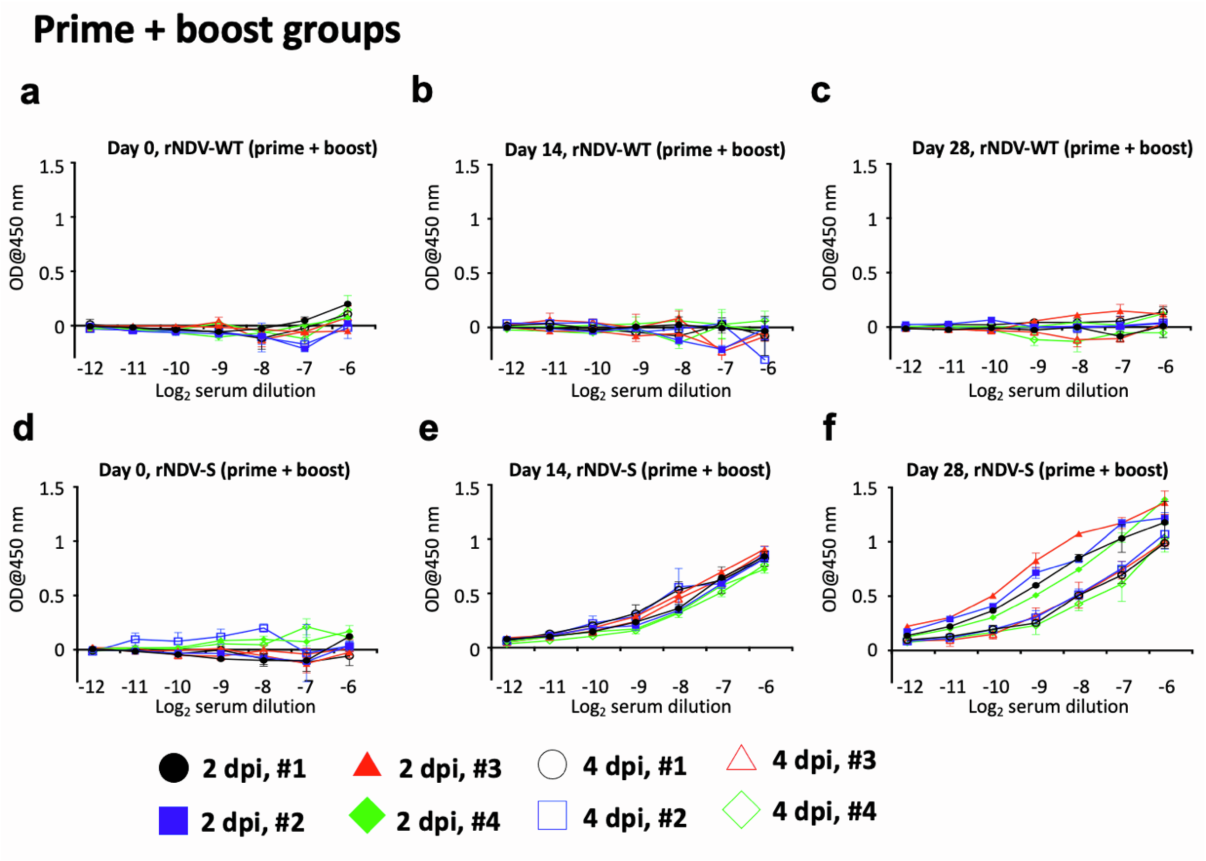

Supplementary Figure 9. Total Abs in serum of individual golden Syrian hamsters vaccinated with rNDV constructs, Related to Figure 6. Levels of total S binding Abs in sera from individual hamsters vaccinated with either rNDV-WT at 0 (**a**), 14 (**b**) and 28 (**c**) days. Levels of total S binding Abs in sera from individual hamsters vaccinated with either rNDV-rNDV-S at 0 (**d**), 14 (**e**) and 28 (**f**) days DPV (prime+boost). Individual hamster titres of Abs are displayed after 2 DPI and 4 DPI. The average of Abs titre in each group were presented in the Figure 6 in the main manuscript.

Supplementary Figure 10, Related to STAR Method: Sequence of the SARS-CoV-2 used in the virus challenge study. The red sequence highlights the cleavage site (^{680}SPRRAR↓SV^{687}).

```
MFVFLVLLPLVSSQCVNLTTRTQLPPAYTNSFTRGVYYPDKVFRSSVLHSTQDLFLPFFSNVTWFHAIHV
SGTNGTKRFDNPVLPFNDGVYFASTEKSNIIRGWIFGTTLDSKTQSLLIVNNATNVVIKVCEFQFCNDPF
LGVYYHKNNKSWMESEFRVYSSANNCTFEYVSQPFLMDLEGKQGNFKNLREFVFKNIDGYFKIYSKHTPI
NLVRDLPQGFSALEPLVDLPIGINITRFQTLLALHRSYLTPGDSSSGWTAGAAAYYVGYLQPRTFLLKYN
ENGTITDAVDCALDPLSETKCTLKSFTVEKGIYQTSNFRVQPTESIVRFPNITNLCPFGEVFNATRFASV
YAWNRKRISNCVADYSVLYNSASFSTFKCYGVSPTKLNDLCFTNVYADSFVIRGDEVRQIAPGQTGKIAD
YNYKLPDDFTGCVIAWNSNNLDSKVGGNYNYLYRLFRKSNLKPFERDISTEIYQAGSTPCNGVEGFNCYF
PLQSYGFQPTNGVGYQPYRVVVLSFELLHAPATVCGPKKSTNLVKNKCVNFNFNGLTGTGVLTESNKKFL
PFQQFGRDIADTTDAVRDPQTLEILDITPCSFGGVSVITPGTNTSNQVAVLYQDVNCTEVPVAIHADQLT
PTWRVYSTGSNVFQTRAGCLIGAEHVNNSYECDIPIGAGICASYQTQTNSPRRARSVASQSIIAYTMSLG
AENSVAYSNNSIAIPTNFTISVTTEILPVSMTKTSVDCTMYICGDSTECSNLLLQYGSFCTQLNRALTGI
AVEQDKNTQEVFAQVKQIYKTPPIKDFGGFNFSQILPDPSKPSKRSFIEDLLFNKVTLADAGFIKQYGDC
LGDIAARDLICAQKFNGLTVLPPLLTDEMIAQYTSALLAGTITSGWTFGAGAALQIPFAMQMAYRFNGIG
VTQNVLYENQKLIANQFNSAIGKIQDSLSSTASALGKLQDVVNQNAQALNTLVKQLSSNFGAISSVLNDI
LSRLDKVEAEVQIDRLITGRLQSLQTYVTQQLIRAAEIRASANLAATKMSECVLGQSKRVDFCGKGYHLM
SFPQSAPHGVVFLHVTYVPAQEKNFTTAPAICHDGKAHFPREGVFVSNGTHWFVTQRNFYEPQIITTDNT
FVSGNCDVVIGIVNNTVYDPLQPELDSFKEELDKYFKNHTSPDVDLGDISGINASVVNIQKEIDRLNEVA
KNLNESLIDLQELGKYEQYIKWPWYIWLGFIAGLIAIVMVTIMLCCMTSCCSCLKGCCSCGSCCKFDEDD
SE PVLKGVKLHYT
```

Supplementary Table1. Summary of NT_{50} values of neutralization assays, Related to Figure 6.

Serum collection day	Experimental group	Average of NT_{50} with pre-treatment (folds dilution)	Average of NT_{50} with post-treatment (folds dilution)
0 DPV	rNDV-WT (prime)	< 100	< 100
0 DPV	rNDV-WT (prime + boost)	< 100	< 100
0 DPV	rNDV-S (prime)	< 100	< 100
0 DPV	rNDV-S (prime + boost)	< 100	< 100
14 DPV	rNDV-WT (prime)	< 100	< 100
14 DPV	rNDV-WT (prime + boost)	< 100	< 100
14 DPV	rNDV-S (prime)	< 100	< 100
14 DPV	rNDV-S (prime + boost)	< 100	< 100
28 DPV	Mock-vaccinated	< 100	< 100
28 DPV	rNDV-WT (prime + boost)	< 100	< 100
28 DPV	rNDV-S (prime + boost)	309.38	102.43
